# Comparative Advances in Sulfide and Halide Electrolytes for Commercialization of All‐Solid‐State Lithium Batteries

**DOI:** 10.1002/adma.202513255

**Published:** 2025-09-28

**Authors:** Mohamed Djihad Bouguern, Ningaraju Gejjiganahalli Ningappa, Karthik Vishweswariah, Anil Kumar M R, Ryoji Kanno, Karim Zaghib

**Affiliations:** ^1^ Department of Chemical and Materials Engineering Concordia University Montreal QC H3G 1M8 Canada; ^2^ Research Center for All‐Solid‐State Battery Institute of Integrated Research Institute of Science Tokyo 4259 Nagatsuta Midori‐ku Yokohama 226‐8502 Japan

**Keywords:** all‐solid‐state lithium batteries (ASSBs), halide electrolytes, interfacial stability, ionic conductivity, sulfide electrolytes

## Abstract

All‐solid‐state lithium batteries (ASSBs) outperform lithium‐ion batteries (LIBs) in safety, energy density, and thermal stability. Their performance depends on high ionic conductivity, chemical/physical stability, and scalable manufacture of solid electrolytes (SEs). This study compares sulfide‐ and halide‐based SEs, two promising next‐generation energy storage options. Soft mechanics permit sulfides with high room‐temperature conductivity, low activation energies, and processability, but high‐voltage cathode instability, moisture sensitivity, and probable hydrogen sulfide (H_2_S) release. Market prospects are favorable as the industry improves crystallinity and elemental substitution, especially for automotive cells. Chloride‐based halides are more environmentally friendly, have adequate voltage stability, and can be used with oxide cathodes without coatings. Despite traditionally low conductivity, high‐entropy, and oxyhalide chemistries currently reach 10 mS cm^−1^, and scalable solvent syntheses and dry processing are driving adoption. Mechanical compliance and the use of rare elements (In, Sc) continue to cause integration and cost issues. Composition, microstructure, synthesis techniques, interfacial behavior, mechanical characteristics, and scalability are evaluated. The findings show sulfides have better conductivity and Li‐metal compatibility, but halides are more stable and manufacturable, recommending hybrid or tailored material selection based on application. Optimizing ASSB systems requires complementary sulfide/chloride utilization due to halides' mechanical constraints.

## Graphical abstract

This review compares sulfide‐ and halide‐based solid electrolytes for all‐solid‐state lithium batteries (ASSBs), highlighting their ionic conductivity, chemical stability, manufacturability, and compatibility with lithium metal. Sulfides offer higher conductivity, while halides provide enhanced stability and scalability. The study emphasizes hybrid strategies, interfacial design, and material selection to enable safer, high‐performance, and commercially viable next‐generation solid‐state batteries.

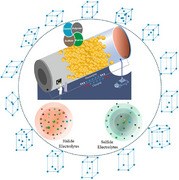

## 1 Introduction

In 1800, Alessandro Volta created the Voltaic Pile, marking the birth of electrochemical batteries, in which he stacked layers of Zn, Cu, and saltwater‐soaked paper to create a simple yet effective battery that produced a steady electric current. This invention set the stage for future battery advancements, showing that chemical reactions can generate electricity.^[^ ^1^ ^]^ Solid‐state ionics traces its roots to Michael Faraday's pioneering work in the 19th century. His research in electrochemistry, especially on electrolysis and ion movement, laid the groundwork for developing solid electrolytes (SEs).^[^ ^2^ ^]^ In 1968, David V. Ragone revolutionized electric vehicles and battery technology. His publication highlighted the need to optimize both energy storage and power delivery for efficient performance. This emphasis significantly influenced the development of electric vehicle technology. Practical applications include increasing driving range, improving acceleration, and enhancing overall vehicle efficiency.^[^ ^3^ ^]^ In 1991, Sony launched Li‐ion batteries (LIBs),^[^ ^4^ ^]^ which provided a significant leap in energy density and rechargeability, making them ideal for smartphones and electric cars. Advancements in LIB technology have extended battery life, increased charging speed, and improved safety, securing their dominance in the market. Today, LIBs are crucial for modern energy storage, powering gadgets, and large renewable energy systems.^[^ ^5^ ^]^ Solid‐state batteries (SSBs) are a significant advancement in battery technology because they offer greater safety, higher energy density, and improved thermal stability compared to liquid‐electrolyte batteries. These benefits arise from SEs, which can be made of polymers, ceramics, or glasses, each contributing unique qualities to the battery performance.^[^ ^6^, ^7^ ^]^ Polymer electrolytes, with a transport number between 0.2 and 0.5, improve ion transport efficiency and are vital for high‐power SSBs, ideal for electric vehicles and portable electronics. They can also be customized with mechanical properties for rugged environments.^[^ ^8^, ^9^ ^]^ Ceramic electrolytes usually have high cationic transference numbers, often close to 1, and are valued for their strong thermal and chemical stability, which improves battery safety and longevity. The high ionic conductivity of ceramic‐doped, cross‐linked solid polymer electrolytes suggests high potential for high‐performance batteries.^[^ ^10^, ^11^ ^]^ Glass SEs typically comprise network‐forming oxide (or sulfide) bases like SiO_2_ or P_2_S_5_ in addition to other oxides or sulfides that modify this network, such as Li_2_O and Li_2_S, respectively.^[^ ^12^, ^13^, ^14^ ^]^ Nevertheless, these electrolytes suffer from shortcomings such as low ionic conductivity, mechanical brittleness, and high interfacial resistance that suppress/enhance their performance in SSBs.^[^ ^15^, ^16^, ^17^ ^]^ Glass‐ceramic SEs, such as those made from Li_2_S‐P_2_S_5_, provide strong mechanical strength and thermal stability. They help prevent dendrite growth and thermal runaway, ensuring the safe operation of Li‐ and Na‐ion batteries.^[^ ^18^ ^]^ SEs can be categorized based on structure (polymer, ceramic, or glass) and chemical composition. Many ceramics and glasses belong to the sulfide or halide families, which are also structural groups. For instance, sulfides can be crystalline ceramics, glasses, or glass‐ceramics, while halides are usually crystalline ceramics. This discussion focuses on sulfide and halide electrolytes as two of the most promising yet contrasting chemistries for next‐generation SSBs. This study examines the function of halide and sulfide SEs in LIBs and beyond. Sulfides are excellent candidates for use in SSBs owing to their high ionic mobility and capacity to create stable surfaces. However, because of their extreme sensitivity to moisture, they gradually deteriorate. Conversely, halides have lower ionic conductivity and require more complicated manufacturing procedures; however, they are also significantly more stable and effectively resist moisture. This study compares the disadvantages and advantages of the two materials and delves into their potential to impact battery technology in the future, considering both electrochemical and chemical stability.

## 2 General Properties and Requirements for Electrolytes in LIBs and SSB

**Figure 1:**
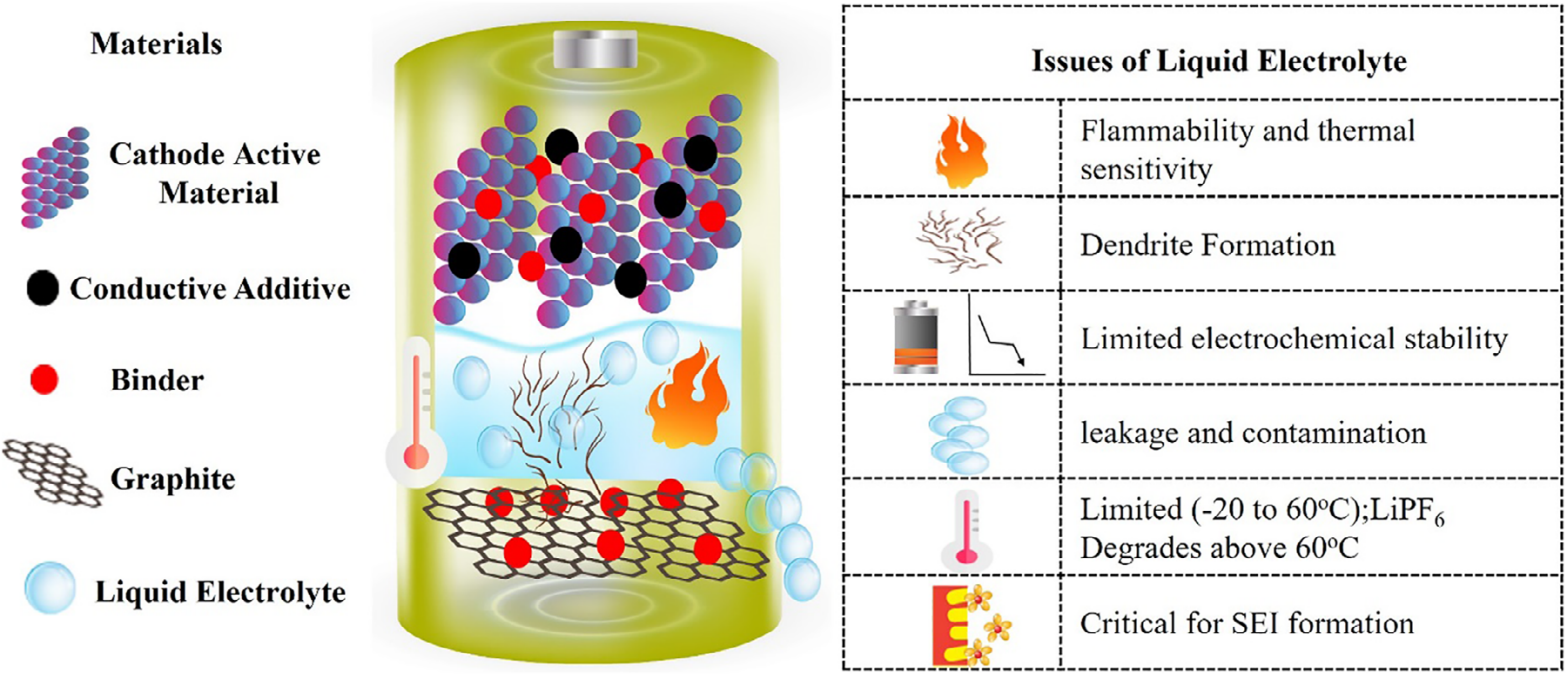
Challenges of Liquid Electrolytes in Lithium‐Ion Batteries.

The electrolyte is one of the most important components among all the parts in LIBs and facilitates the transportation of Li ions between the anode and cathode during the charging/discharging cycle. The liquid electrolyte used is typically a Li salt, such as LiPF_6_, dissolved in organic solvents like ethylene carbonate (EC) or dimethyl carbonate (DMC). These solvents offer high ionic conductivity, allowing for a broader electrochemical stability window with good compatibility with both electrodes. It could be a thermally stable and non‐toxic electrolyte, in the best case.^[^ ^19^ ^]^ However, most organic electrolytes are flammable, a major safety hazard that the current trends seem unlikely to solve,^[^ ^20^, ^21^ ^]^ as shown in **Figure** **1**.

**Figure 2:**
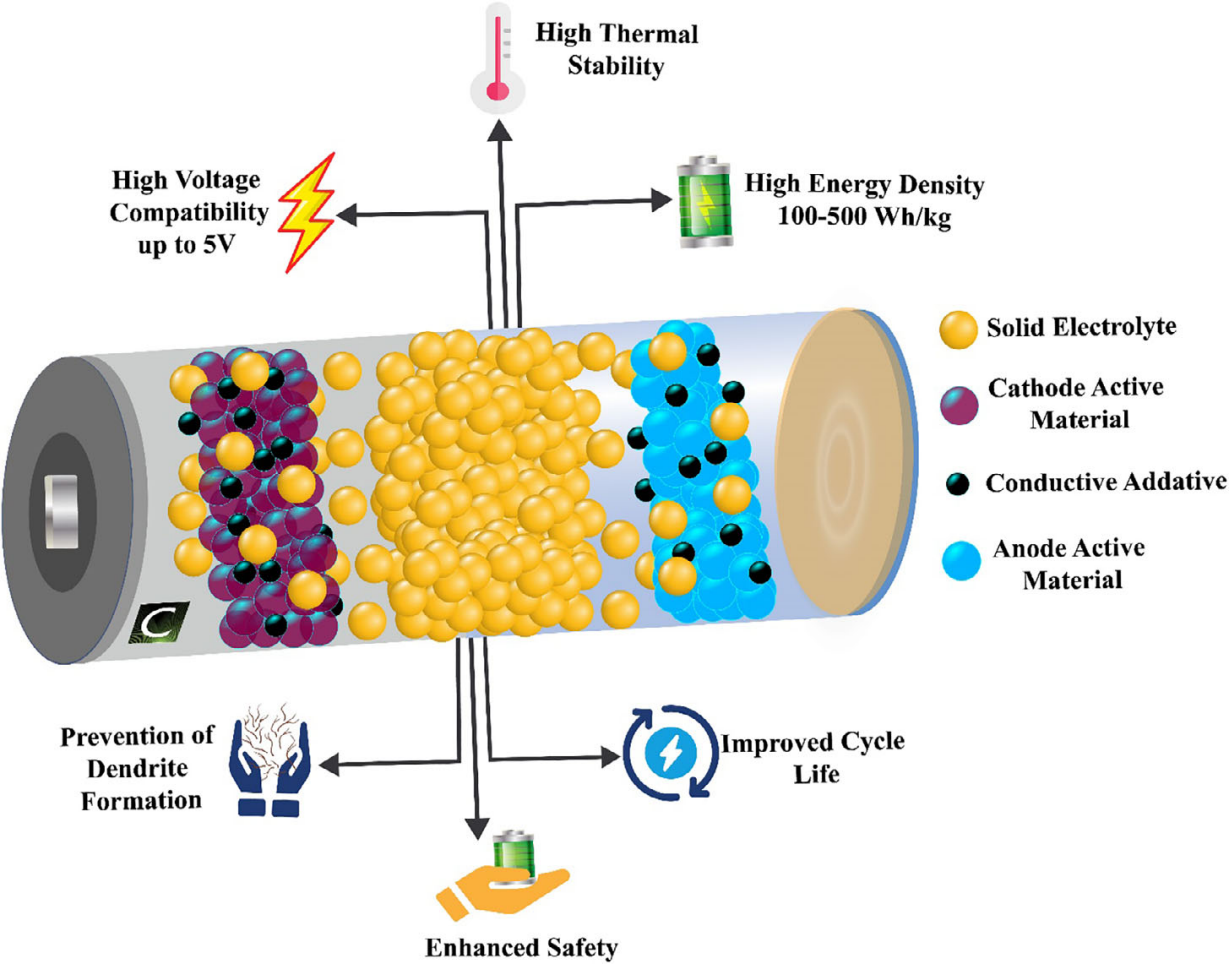
Advantages of Solid‐State Electrolytes in Next‐Generation Batteries.

The legacy of Michel Armand and his earliest work with polymer electrolytes helped make solid‐state Li batteries (SSBs) possible. SSBs can overcome some of the limitations currently faced by traditional liquid electrolyte Li‐ion cells and use a solid electrolyte for faster ion conduction. This significantly improves the safety by eliminating flammable liquid electrolytes and allows for greater energy densities to be achieved.^[^ ^22^ ^]^ In this context, SEs can be classified by structure/morphology (polymers, ceramics, glasses, or glass‐ceramics) or by chemical composition (sulfide‐based, halide‐based, oxide‐based, etc.). For high‐performance applications in SSBs, SSEs must combine several essential properties: high ionic conductivity at room temperature, low electronic conductivity to prevent short circuits, wide electrochemical stability windows, mechanical strength to suppress Li dendrite growth, and chemical stability with electrode materials.^[^ ^6^, ^23^ ^]^ Achieving high ionic conductivity while maintaining good electrode contact remains a key challenge. Other important requirements include mechanical flexibility for scalable manufacturing, stability across a wide operating temperature range (−40 to +50 °C for commercial use), and compatibility with diverse battery architectures^[^ ^24^, ^25^ ^]^ (**Figure** **2**).

**Table** **1** summarizes the unique properties of both LIB and SSB electrolytes. They should have low electronic conductivity to prevent short circuits, demonstrate a wide operating temperature window without undergoing stability issues between −40 and +50 °C, and enable fast Li‐ions transport with no or minor side reactions. Mechanical flexibility and ease of processing are also necessary for commercial scalability in the SSBs.

**Table 1:** Comparison of liquid and solid‐state electrolytes for Li‐Ion batteries.

Property	LIB electrolytes (liquid)	SSB electrolytes (solid)	Ref
Material used	Organic solvents, Li salts, additives (e.g., LiPF_6_, LiBF_4_, LiTFSI, LiFSI), solvents (e.g., EC, DMC, DEC)	Ceramics (e.g., Li_7_La_3_Zr_2_O_12_), sulfides (e.g., Li_10_GeP_2_S_12_), halides (e.g., Li_3_InCl_6_, Li_3_YCl_6_), polymers (e.g., PEO)	[19, 26, 27, 28, 29]
Voltage window	0–4.5 V	Up to 5 V (ceramics, halides are better for high voltage)	[30, 31]
Energy density	150–250 Wh kg^−1^	300–500 Wh kg^−1^	[32, 33, 34]
Structure	Liquid with dissolved Li salts; low viscosity	Solid, crystalline, or amorphous, acts as both electrolyte and separator	[21, 35, 36]
Thermal stability	Limited (–20–60 °C); LiPF_6_ degrades above 60 °C.	High thermal stability (>125 °C for ceramics, halides)	[21, 37, 38, 39, 40]
Interface engineering	SEI formation critical	Solid‐solid interface, pressure or coatings needed.	[41, 42, 43]
Transport of Li	Fast Li transport due to the liquid medium	Solid electrolytes transport Li more slowly	[42, 44, 45]
Mechanical properties	Not critical; handled by separator	Critical; must maintain contact between electrolyte and electrodes	[46, 47, 48]
Mechanical flexibility	High flexibility: liquid electrolytes allow easy flow and conform to the battery shape	Low flexibility for ceramics and sulfides; polymer electrolytes offer moderate flexibility	[49, 50]
Dendrite suppression	Dendrites may form, requiring additives	Better suppression, but still possible in some materials	[41, 51, 52]
Safety	Flammable, risk of leakage, and thermal runaway	Non‐flammable, reduced risk of thermal runaway	[53, 54, 55]

Electrolytes for LIB and SSB must exhibit high ionic conductivity to facilitate efficient Li‐ion transport at RT alongside a wide electrochemical stability window to support operation across broad voltage ranges. They require excellent thermal stability to withstand a wide range of temperatures, particularly at higher levels. Ideally, they must be non‐combustible to enhance protection with the prevention of fire and thermal “runaway.” Additionally, good mechanical properties are essential to suppress Li dendrite formation and maintain solid contact with electrodes. Chemical stability is necessary for inhibiting degradation or side reactions with the electrode materials and dictates appropriate matches of anode/cathode potential. Critically, as well, the electrolyte needs to help manufacturers scale and be designed for incorporation with different battery architectures.

## 3 Sulfide Electrolytes

**Figure 3:**
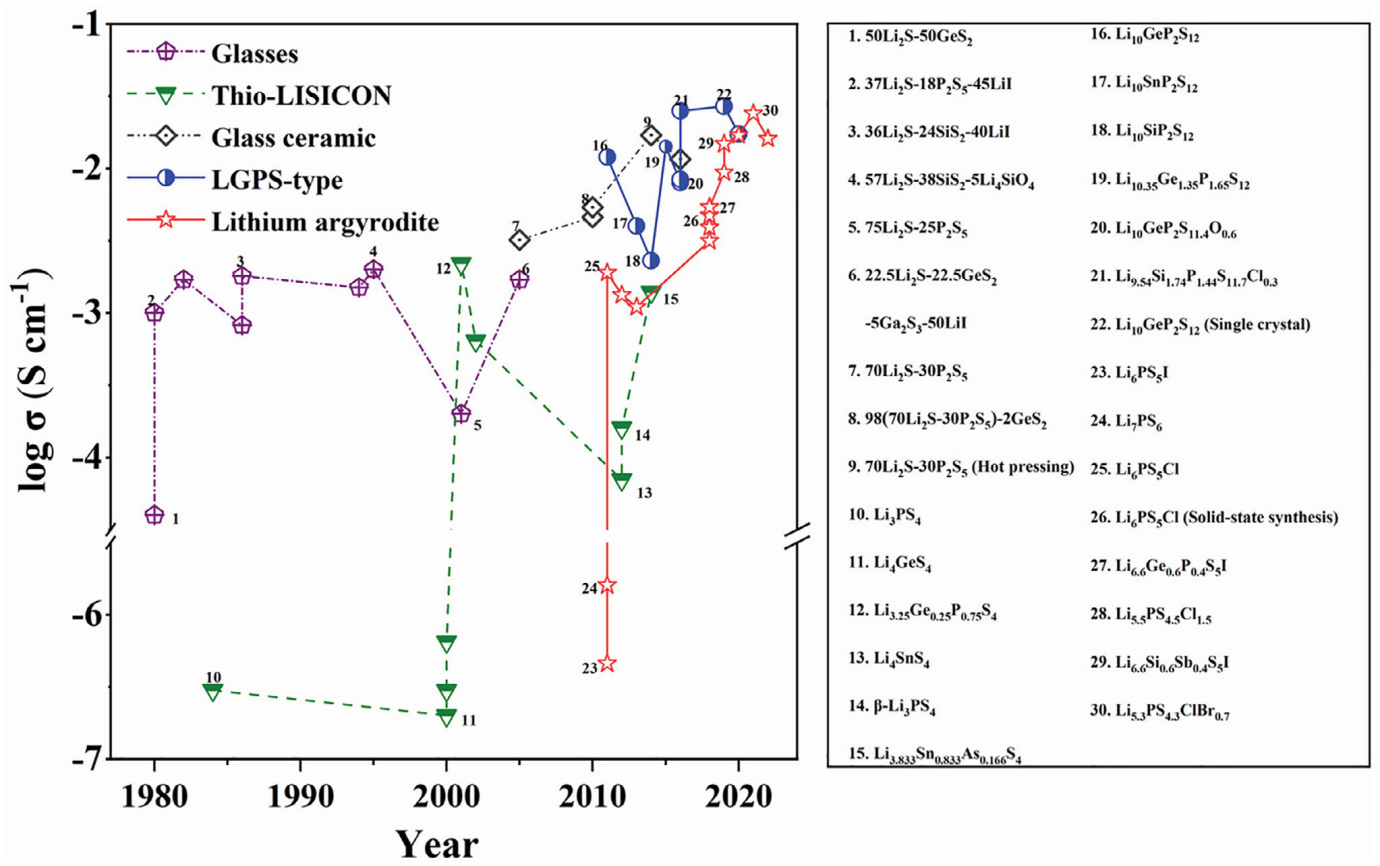
Chronological account of developments in sulfide ionic conductors, including the room‐temperature conductivity and publication year of representative material groups. Adapted with permission.^[^ ^61^ ^] Copyright 2023, Wiley‐VCH GmbH.^

In the field of high‐performance all‐solid‐state Li batteries (ASSBs), sulfide SEs, mainly based on Li and sulfur compounds, have offered promising prospects for a comprehensive application. With ionic conductivity approaching that of traditional liquid electrolytes, Li‐ion transport is found to be equally effective. It is more efficient owing to the polarizability of sulfur atoms, which increases Li‐ion mobility over that achievable in oxygen‐based electrolytes.^[^ ^56^, ^57^ ^]^ Furthermore, sulfide electrolytes possess outstanding mechanical properties such as flexibility and ductility, supporting the cost‐effective manufacturing of dense, low‐resistance layers via cold‐pressing techniques at relatively mild temperatures.^[^ ^58^, ^59^ ^]^ However, the stability and reactivity of these materials are limited by their sensitivity to moisture, which can result in the release of toxic hydrogen sulfide (H_2_S) gas, as well as compatibility issues with certain electrode materials, While sulfide‐based batteries have already found commercial use in small‐scale applications such as Maxell's chip batteries used in industrial machinery further improvements in chemical stability and interfacial compatibility will be essential for their broader deployment in large scale energy storage systems.^[^ ^60^ ^]^ As illustrated in **Figure** **3**, the development of sulfide electrolytes has evolved significantly over time. Originally focused on simple binary systems, the trend now emphasizes the creation of more complex materials designed to enhance the chemical stability and ionic conductivity.

### 3.1 Composition and Structure

The composition of sulfide electrolytes includes a blend of Li salts, S, and additional elements such as P, Si, Ge, and Sn, or halogens such as Cl, Br, and I. These electrolytes are formulated to improve the conductivity and stability of Li‐ion in ASSBs. An elaborate description of the components of the primary sulfide electrolyte systems is given below.

#### 3.1.1 Thio‐LISICON

The Li superionic conductor (LISICON), specifically Li_14_Zn(GeO_4_)_4_, is an example of a material designed using crystallographic principles underlying fast alkali‐ion transport.^[^ ^62^ ^]^ The thio‐LISICON crystalline material family was discovered in the Li_2_S‐GeS_2_‐P_2_S_5_ system by Kanno and Murayama in 2001. Their findings revealed high ionic conductivity in the newly identified thio‐LISICON, Li_4‐2x_Ge_1‐2x_P_x_S_4_ (LGPS). This material shows excellent electrochemical stability, no reactivity with Li metal, and no phase transitions up to 500 °C.^[^ ^63^, ^64^ ^]^

#### 3.1.2 Argyrodite

Argyrodite (Ag_8_GeS_6_), a mineral made up of Ag, Ge, and S, was discovered by Clemens Winkler in 1885.^[^ ^65^ ^]^ The name of this mineral originates from the Greek word for silver, “argyros.” Solid‐state chemistry and materials researchers have paid considerable interest to the mineral because of its unusual crystal structure. The discovery of sulfide‐based SEs, motivated by the argyrodite structure, marked a significant breakthrough in the development of SSBs. Early in the new millennium, scientists discovered that it might improve ion transport, especially for Na and Li ions. Since then, a great deal of work has gone into enhancing stability and ionic conductivity, two crucial factors for the advancement of SSB technology. Due to their remarkable Li‐ion conductivity, Li argyrodites, such as Li_6_PS_5_X (where X indicates elements like Cl, Br, or I), have attracted special attention.^[^ ^7^, ^66^ ^]^

#### 3.1.3 Glass‐Ceramic

Using glass electrolytes, Dr. Kondo made the first attempt to create a sulfide‐based battery that aligns with current research trends. He was able to develop an electrolyte that enabled a battery architecture similar to that of LIBs in a sulfide‐based system by adding trace amounts of phosphate (PO_4_) to a Li‐P‐S glass matrix, thereby improving chemical stability.^[^ ^67^ ^]^ Researchers are attempting to develop SEs that combine the best features of glass, glass‐ceramic, and crystalline conductor systems. Glassy electrolytes, such as Li_2_S‐P_2_S_5_, are more versatile and can accommodate different additions and dopants. Conversely, glass ceramics provide a compromise between the high conductivity of crystalline materials and the processing simplicity of glasses. A major advancement in solid‐state electrolyte development, especially for LIBs, is represented by the Li_2_S‐P_2_S_5_ glass‐ceramic family. Leading candidates for next‐generation battery technologies, these materials combine high ionic conductivity, improved electrochemical stability, and the capacity to generate superionic crystals from precursor glasses.^[^ ^68^, ^69^ ^]^

#### 3.1.4 LPS

**Figure 4:**
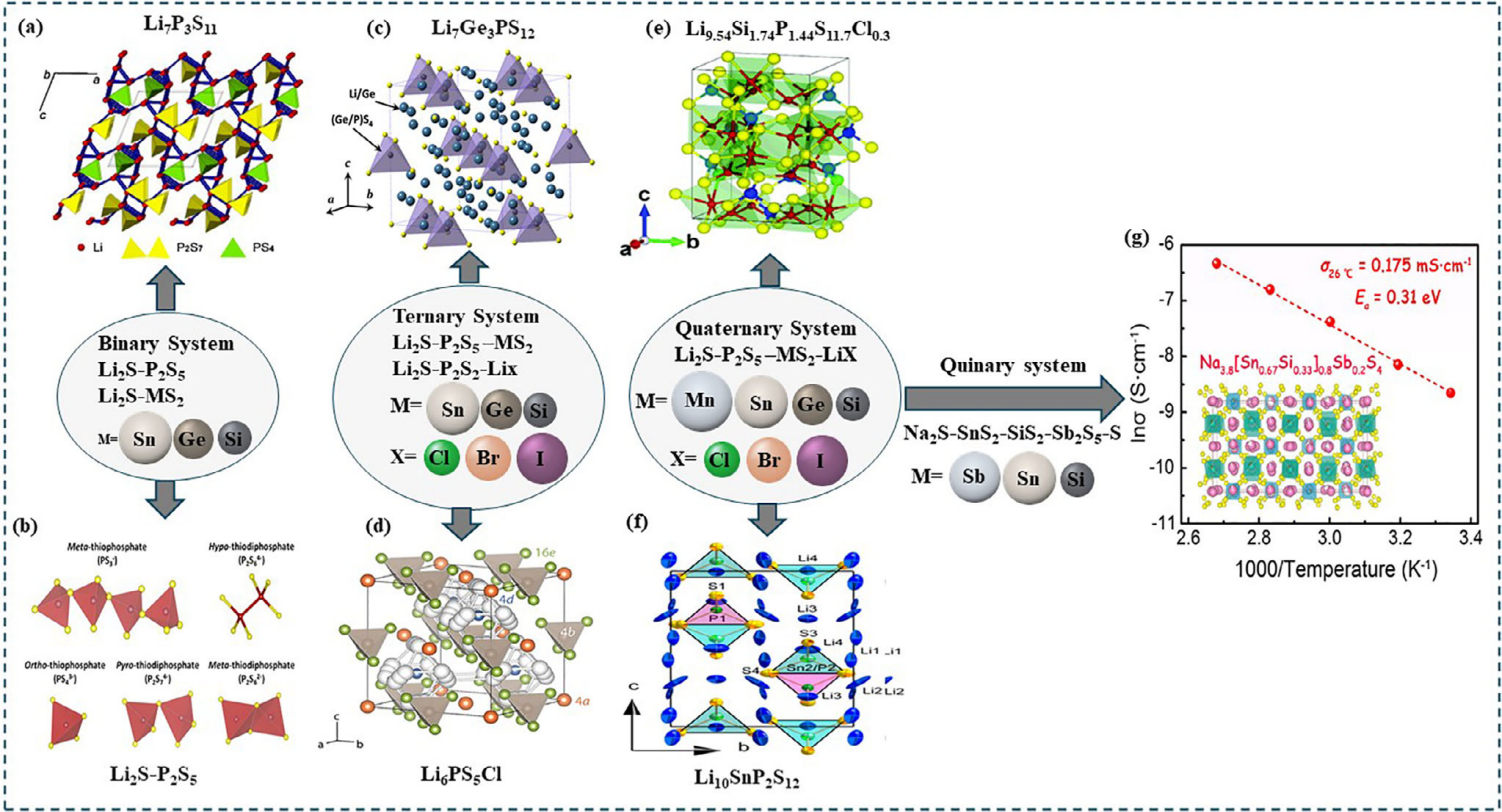
Structural forms of SEs and their representative examples: a) The structural representation of the superionic Li_7_P_3_S_11_ crystal in a binary system. Adapted with permission.^[^ ^75^ ^] Copyright 2013, Elsevier;^ b) The anionic species found in materials produced within the Li_2_S‐P_2_S_5_ binary system. Adapted with permission.^[^ ^85^ ^]^ Copyright 2018, Elsevier; c) The crystal structure of the argyrodite‐type Li_7_Ge_3_PS_12_ in a ternary system. Adapted with permission.^[^ ^76^ ^]^ Copyright 2016, Elsevier; d) The argyrodite‐type Li_6_PS_5_X crystal structure exhibiting cubic symmetry in the space group F43m. Adapted with permission.^[^ ^77^ ^]^ Copyright 2019, American Chemical Society; e) The crystal structure of typical Li_9.54_Si_1.74_P_1.44_S_11.7_Cl_0.3_ in the context of a quaternary system. Adapted with permission.^[^ ^78^ ^]^ Copyright 2021, Royal Society of Chemistry; f) The crystal structure of Li_10_SnP_2_S_12_within a quaternary system. Adapted with permission.^[^ ^79^ ^]^ Copyright 2013, American Chemical Society; g) The crystal structure of Na_3.8_[Sn_0.67_Si_0.33_]_0.8_Sb_0.2_S_4_ within a quinary system. Adapted with permission.^[^ ^80^ ^] Copyright 2019, Elsevier and Science Press.^

ASSBs make extensive use of the Li_3_PS_4_ family of sulfide‐based SEs due to their soft mechanical qualities and high ionic conductivity, which improve the electrode contact and lower interfacial resistance.^[^ ^70^ ^]^ In order to overcome scalability issues, studies have focused on enhancing its conductivity and stability through element doping with halogens or oxides and improving synthesis techniques.^[^ ^71^ ^]^ Li_3_PS_4_, which is synthesized by the reaction of Li_2_S and P_2_S_5_, has PS_4_^3−^ units that help transport Li ions.^[^ ^72^ ^]^ When doped with halides like LiBr, which enhance ion mobility and shift ion transport from 2D to 3D, it performs exceptionally well in its β‐phase.^[^ ^73^, ^74^ ^]^ Due to these developments, Li_3_PS_4_ has become a crucial component in SSBs, which are safer and more effective. The development of SSBs depends on each structural shape, as illustrated in **Figure** **4**, which is designed to improve ionic mobility, lower grain boundary resistance, and increase the electrode compatibility. SSE materials are based on binary systems, as seen in Figure 4a,b; however, these systems often have poor chemical stability and lower ionic conductivity. Moving on to ternary systems, as shown in Figure 4c,d, allows for substantial increases in ionic conductivity beyond 10^−3^ S cm^−1^ and provides chances for structural optimization by incorporating dopants. By combining many elemental components, further development into quaternary systems, as shown in Figure 4e,f, allows for a more accurate adjustment of structure, ion transport routes, and electrochemical stability. Lastly, quinary systems emphasize scalability and the utilization of earth‐abundant elements, such as the Na_3_SbS_4_‐based materials depicted in Figure 4g, which broaden the design potential toward sodium‐ion batteries.

By default, Ionic conductivity testing is conducted at RT unless noted otherwise.

A comprehensive strategy that emphasizes crystalline structure and composition adjustment is needed to improve the ionic conductivity and stability of sulfide‐based SEs in ASSBs, as shown in **Table** **2**. Such strategies include stabilizing surface and grain boundaries and optimizing crystalline structure and composition modifications, and element selection. These techniques also aid in material stabilization, promote structural stability, decrease grain boundary resistance, ameliorate high voltage decomposition problems, and avoid the formation of Li dendrites.^[^ ^108^, ^109^, ^110^ ^]^

**Table 2:** The structures and conductivities of typical binary and ternary sulfide solid electrolytes.

Family	Composition	Structure	Ionic conductivity S cm^−1^	Element selection	Refs.
Li_7_P_3_S_11_ series	Li_7_P_3_S_11_ Crystalline Li_7_P_3_S_11_ Ball‐milled	Triclinic Triclinic	1.57 × 10^−4^ at RT 2.8 × 10^−^^3^ ^−3^ at 60 °C 4.3 × 10^−5^ 3.6 × 10^−4^ at 60 °C	–	[81]
Li_7_P_3_S_11_ series	Li_7_P_3_S_11_	Triclinic	3.2 × 10^−3 ^	–	[82]
Li_7_P_3_S_11_ series	Li_7_P_3_S_11_ Li_7_P_3_S_11_‐_Z_ (modified version, substitution of P_2_S_3_ for P_2_S_5_	Triclinic Triclinic	3.2 × 10^−3^ 5.4 × 10^−3^	P_2_S_3_ substituted for P_2_S_5_	[83]
LPS	β‐Li_3_PS_4_	Orthorhombic	1.6 × 10^−4^	–	[84, 85]
Thio‐LISICON	Li_4_GeS_4_ Li_4‐2x_Zn_x_GeS_4_ Li_4+x+δ_(Ge_1−δ′−x_–Ga_x_)S_4_	Orthorhombic Orthorhombic Orthorhombic	2.0 × 10^−7^ 3.0 × 10^−7^ 6.5 × 10^−5^	Zn, Ga	[86]
Thio‐LISICON	2Li_2_S–1SnS_2_	Orthorhombic	7 × 10^−5^ at 20 °C	Sn	[87]
Argyrodite	Li_6_PS_5_Br	Cubic	2.58 × 10^−3^	Br	[88]
Argyrodite	Li_6_PS_5_Cl/ P(VDF‐TrFE)	Cubic	1.20 × 10^−3^	Cl, polymer	[89]
Argyrodite	Li_6_PS_5_Cl_0.7_I_0.3_	Cubic	2.33 × 10^−3^	Cl, I	[90]
Argyrodite	Li_6.6_P_0.8_Sn_0.2_S_5_I_0.6_Cl_0.4_	Cubic	9.6 × 10^−4^	Sn, I, Cl	[91]
Argyrodite	Li_9.95_SnP_2_S_11.95_F_0.05_	Tetragonal	6.4 × 10^−3^	Sn, F	[92]
Argyrodite	Li_9.54_Si_1.74_P_1.44_S_11.7_Cl_0.3_	Tetragonal	2.5 × 10^−2^	Si, Cl	[7]
Argyrodite	Li_6+x_P_1‐x_Si_x_S_5_I (where 0 ≤ x ≤ 0.50)	Cubic	7.34 × 10^−3^	Si	[93]
Glass‐Ceramic	t‐Li_7_SiPS_8_	Tetragonal	5.3‐6.6 × 10^−3^ (depending on pressure and particle size)	Si	[94]
Glass‐Ceramic	Li_7_SiPS_8_	Tetragonal Orthorhombic	2 × 10^−3^ 0.13 × 10^−3^	Si	[95]
Argyrodite	Li_6_PS_5_Cl Li_6_PS_5_Cl‐0.05SnI	Cubic Cubic	1.9 × 10^−3^ 3.9 × 10^−3^	Sn, I	[96]
Li‐Si‐S System	Li_2_SiS_3_ Li_4_SiS_4_	Orthorhombic Orthorhombic	2 × 10^−5^ 5 × 10^−8^	Si	[97]
Thio‐LISICON	Li_3.875_Sn_0.875_ As_0.125_S_4_	Orthorhombic	2.45 × 10^−3^	Sn, As	[98]
LGPS	Li_10_GeP_2_S_12_	Tetragonal	1.2 × 10^−2^	Ge	[99]
Li_7_P_3_S_11_ series	Li_7_P_3_S_11_	–	1.7 × 10^−2^	–	[100]
Glass‐Ceramic	Li_5.6_PS_4.6_I_1.4_	–	2.04 × 10^−3^	I	[101]
Argyrodite	Li_6.5_In_0.25_P_0.75_S_5_I	Cubic	1.06 × 10^−3^	In	[102]
Glass‐Ceramic	Li_7_Ag_0.1_P_3_S_11_I_0.1_	Triclinic	1.35 × 10^−3^	Ag, I	[103]
Argyrodit	Li_7_P_2_S_8_I	Tetragonal	1.35 × 10^−3^	I	[104]
LGPS	Li_10_Ge(P_0.925_Sb_0.075_)_2_ S_12_	Tetragonal	1.7 × 10^−2^	Sb	[105]
Thio‐LISICON	Li_11_AlP_2_S_12_	Orthorhombic	8.0 × 10^−4^	Al	[106]
Thio‐LISICON	Li_10.35_[Sn_0.27_Si_1.08_]P_1.65_S_12_	Tetragonal	1.1 × 10^−2^	Sn, Si	[107]

### 3.2 Ionic Conductivity

The capacity of ions to flow through a substance, such as an electrolyte, when exposed to an electric field is known as ionic conductivity (σ). Measured in siemens per meter (S m^−1^), it is essential for battery performance. Effective ion transport is made possible by high ionic conductivity, which improves the performance of energy devices and is influenced by temperature, material composition, and mobile ion concentration. The energy barrier that ions must cross to travel between sites is known as the activation energy (Ea) in this context, and is commonly expressed in electron volts (eV). Particularly at lower temperatures, a lower Ea makes it easier for ions to travel, which increases ionic conductivity. Their relationship is consistent with the Arrhenius equation, which states that higher conductivity results from a decrease in Ea. This is especially true when the temperature rises and, in turn, provides the thermal energy required for ion movement.^[^ ^111^, ^112^, ^113^ ^]^

The ionic conductivity can be described by the Arrhenius equation:

(1) $$\sigma =\sigma_{0}\exp\left(-E_{a}/k_{B}T\right)$$

where σ_0_ is the pre‐exponential factor or the conductivity at infinite temperature, E_a_ is the activation energy for ion migration, k_B_ is the Boltzmann constant, and T is the temperature in Kelvin.

To study ionic conductivity as a function of temperature, we can plot ln(σ) vs 1/T, and obtain a straight line if the process is Arrhenius‐like. The slope of this line gives ‐E_a_/k_B_, and the intercept gives ln(σ_0_).

For sulfide‐based electrolytes, the Nernst‐Einstein equation also helps in relating ionic conductivity to ion mobility:

(2) $$\sigma =\mathrm{nq}\mu \to \sigma =nq^{2}D/k_{B}T$$

where n concentration of the ion, q charge, μ mobility, and D diffusion coefficient.^[^ ^114^, ^115^ ^]^

An experimental method for measuring ionic conductivity is the electrochemical impedance spectroscopy (EIS), which is computed using the formula:

(3) σ=L/RS

where σ, L, R, and S denote the ionic conductivity, thickness of the electrolyte sample, bulk resistance, and area of the electrolyte‐electrode interface, respectively.^[^ ^116^ ^]^

The resistive and capacitive characteristics of materials are frequently analyzed in electrochemistry and battery research using Nyquist plots, which are graphical depictions of complex impedance data. A Nyquist plot for sulfide SEs can provide crucial details on the charge transfer resistance, interface stability, and total ionic conductivity. Degradation processes or interfacial reactions may also be revealed by variations in the Nyquist plot over time, or under various circumstances.^[^ ^117^ ^]^

**Figure 5:**
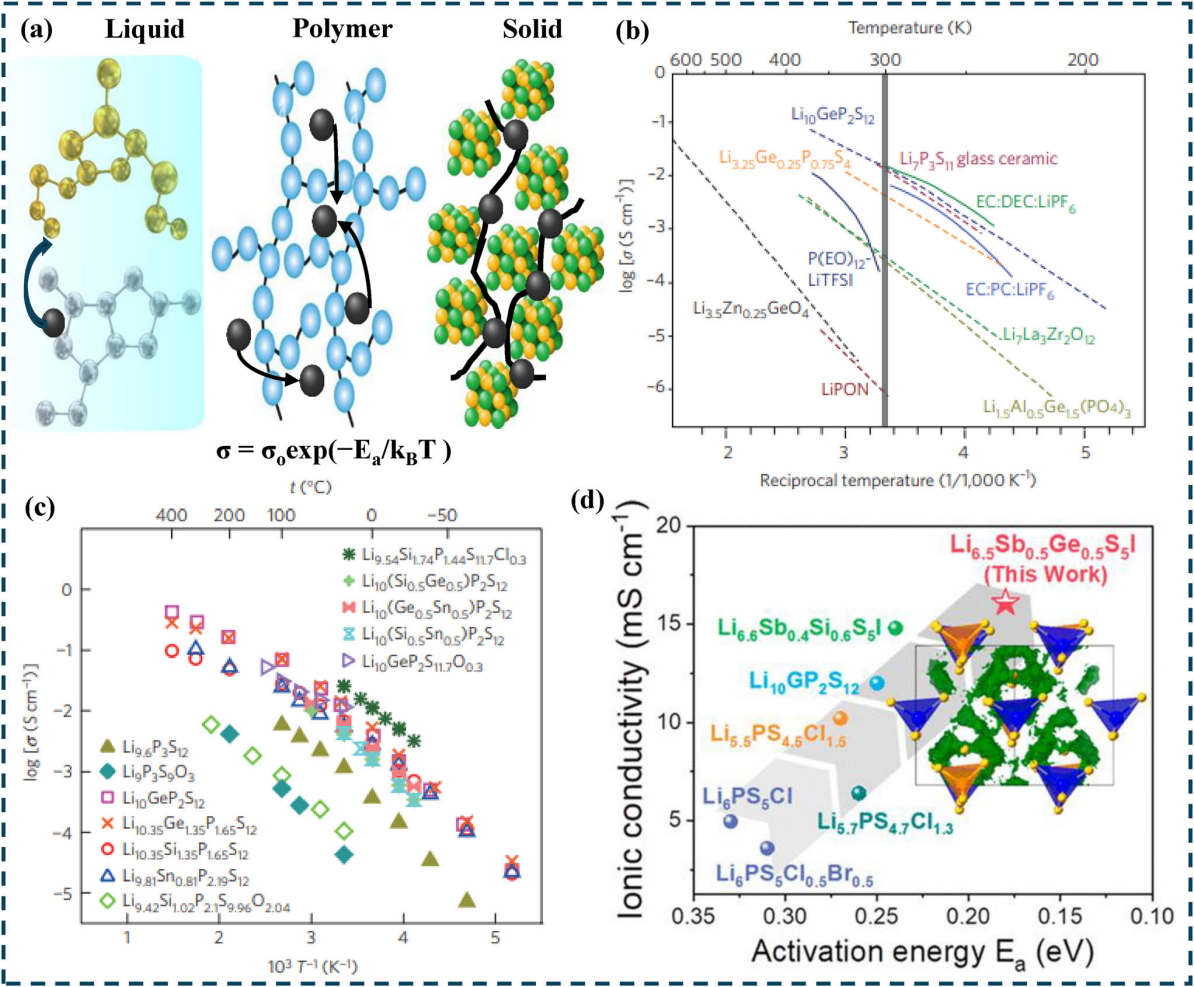
Arrhenius plot of ionic conductivity for: a) Ionic Transport Mechanisms in Liquid, Polymer, and Solid Electrolytes. b) Ionic conductivities of SEs are compared to those of liquid electrolytes and polymer electrolytes. Adapted with permission.^[^ ^122^ ^]^ Copyright 2016, Macmillan Publishers Limited; c) LGPS family and Li _9.6_P_3_S_12_ and Li _9.54_ Si_1.74_ P_1.44_ S _11.7_Cl_0.3_. Adapted with permission.^[^ ^7^ ^]^ Copyright 2016, Springer Nature Limited; d) Ionic Conductivity vs Activation Energy for Various Li Superionic Conductors. Adapted with permission.^[^ ^127^ ^] Copyright 2021, American Chemical Society.^

According to **Figure** **5**a, ions in liquid electrolytes, such as liquid Li salt solutions, freely migrate through a solvent matrix under the influence of concentration gradients and electric fields. SSBs frequently employ polymer electrolytes, which transmit ions by enabling ion hopping between polymer chains, usually at high temperatures, to promote segmental motion. Ions can jump between empty sites in SEs, such as ceramic or glassy materials, because of their rigid lattice structure; the primary foundation of SEs is diffusion along crystallographic channels. The efficiency with which ions or electrons can pass through a material is determined by its molecular structure. This classification draws attention to the variety of ways that various material compositions promote electrical conductivity.^[^ ^118^ ^]^ Cooperative ion movement is typically viewed as beneficial for high ionic conductivity; however, the study by Takeshi et al. on LGPS reveals that it can also have the opposite effect. In tightly packed pathways, strong repulsion between ions forces them to move in a correlated manner, thereby increasing energy barriers and slowing down diffusion. This suggests that, in some cases, cooperative motion may actually suppress ion movement rather than enhance it.^[^ ^119^ ^]^

As illustrated in Figure 5b, some of the highest ionic conductivities are found in sulfide‐based SEs, such as Li_10_GeP_2_S_12_(LGPS),^[^ ^99^ ^]^ Li_3.25_ Ge_0.25_ P_0.75_ S_4,_^[^ ^63^ ^]^ and Li_7_P_3_S_11._^[^ ^100^ ^]^ The steep slope seen in the Arrhenius plot corresponding to these materials demonstrates how the conductivity significantly increases with temperature. The ability of sulfide‐based materials to approach or exceed the liquid electrolyte conductivity, such as EC: DEC: LiPF_6_^[^ ^120^ ^]^ particularly at and above RT, makes them unique among the SSEs. LGPS, for example, has one of the highest conductivities at RT, matching or exceeding that of other polymer electrolytes like P(EO)_12_LiTFSI,^[^ ^121^ ^]^ which are based on polyethylene oxide. LGPS is a good option for SSBs since it combines the robust ionic conductivity of SEs with the inherent safety advantages. Li_7_P_3_S_11_, another glass‐ceramic based on sulfides, also shows excellent conductivity close to RT, though slightly lower than LGPS.^[^ ^122^ ^]^ Figure 5c depicts the Arrhenius plots, which show the temperature‐dependent ionic conductivity (σ) of different solid electrolyte materials. In these plots, the y‐axis denotes the logarithmic scaling of the ionic conductivity (log(σ)), and the x‐axis denotes the inverse temperature (10^3^/T in K^−1^), in accordance with the Arrhenius law, which states that conductivity rises with temperature. The relevant temperatures, which range from ≈−50 to over 400 °C, are also plotted on a supplementary x‐axis at the top for comparison. Owing to the advantageous changes in their lattice structure, high conductivity compositions such as Li_9.54_Si_1.74_P_1.44_S_11.7_Cl_0.3_, Li_10_(Si_0.5_Ge_0.5_)P_2_S_12_, and Li_10_(Ge_0.5_Sn_0.5_P_2_S_12_) show the highest conductivities, especially at lower temperatures. Single‐element substitutions such as Li_10_GeP_2_S_12_^[^ ^123^ ^]^ and Li_10.35_Si_1.35_P_1.65_S_12_^[^ ^124^ ^]^ exhibit good performance in compositions with moderate conductivity, while the dual or triple‐element modifications perform better. Since oxygen atoms impose a more rigid lattice structure, low conductivity compositions such as those including oxygen, like Li_9_P_3_S_9_O_3_^[^ ^125^ ^]^ and Li_9.42_ Si_1.02_P_2.1_S_9.96_O_2.04_^[^ ^126^ ^]^ generally display lower conductivities.

Ionic conductivity is increased by lowering the Ea, which makes it easier for Li ions to flow. In contrast to several other SEs, Li_6.5_Sb_0.5_Ge_0.5_S_5_I achieves lower Ea and higher ionic conductivity (Figure 5d). Further, Li_6_PS_5_I and other earlier argyrodites have lower ionic conductivities (≈0.003 mS cm^−1^) and greater activation energies. By fortifying Ge─S bonds and modifying Li⁺ interactions with the surrounding framework, Ge substitution in this modified argyrodite structure alters the local bonding environment, thereby lowering the diffusion barrier for Li ions. Furthermore, the number of Li ions introduced into high‐energy locations by Ge substitution is higher, thereby supporting “concerted Li‐ion migration.” Such a coordinated ion movement further improves the ionic conductivity.

Sulfide‐based SEs exhibit high ionic conductivity, largely due to the lattice structures, low activation energies, and ion mobility. In structures like LGPS, Thio‐LISICON, and argyrodites, lattice arrangements provide continuous pathways for Li ions, facilitating ion transport. Furthermore, modifications like Ge or Si doping can lower activation energy, allowing ions to move more easily and reducing grain boundary resistance, which is critical for smooth ionic flow. Due to the polarizability of sulfur ions, the “soft” lattice of sulfides further enhances ion mobility by creating a flexible framework that allows ions to hop with less energy. These factors combined give sulfide SEs ionic conductivities at RT that are either equivalent to or higher than those of liquid or polymer electrolytes, making them highly suitable for use in ASSBs.^[^ ^27^ ^]^

### 3.3 Synthesis and Electrochemical Stability

There are several synthesis techniques for sulfide electrolytes, each with its own advantages, disadvantages, and mechanisms. These techniques include mechanochemical synthesis (ball milling), wet chemical synthesis, and solid‐state processes. A brief description of the three techniques is given below.

**Figure 6:**
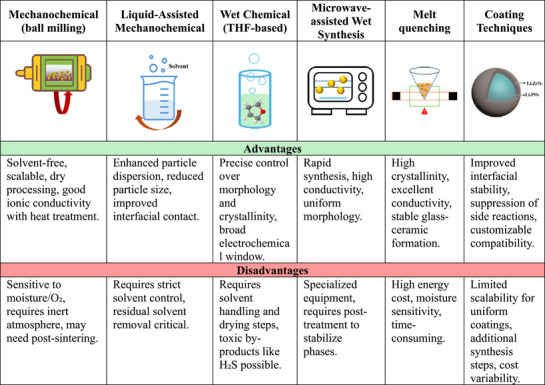
Comparison of synthesis methods for sulfide electrolyte materials: advantages and disadvantages.

i) Mechanochemical synthesis is a scalable, solvent‐free method that uses high‐energy milling to drive reactions between Li_2_S and sulfide precursors like P_2_S_5_ and LiX with (X = Cl, Br, I), offering a dry process but requiring strict control to avoid moisture and oxygen exposure, due to the sulfide's sensitivity to hydrolysis.^[^ ^128^ ^]^ ii) Wet chemical synthesis dissolves or disperses sulfide precursors in organic solvents such as tetrahydrofuran (THF), enabling precise control over particle size and morphology, with the flexibility to produce crystalline or amorphous phases depending on the synthesis conditions, although it requires careful drying and solvent removal to prevent degradation.^[^ ^84^ ^]^ iii) Solid‐state reactions produce highly crystalline phases with superior ionic conductivity by heating solid precursors such as Li_2_S and P_2_S_5_ to high temperatures, frequently above 500 °C. While effective, this method can be sensitive to moisture and may require careful handling, extended processing times, and significant energy input, which can pose challenges for large‐scale manufacturing.^[^ ^129^ ^]^ Mechanochemical methods, though widely used in research, are generally less suitable for industrial‐scale production. In contrast, liquid‐phase synthesis offers a more practical and scalable approach for manufacturing, making it particularly attractive from an industrial perspective, as shown in **Figure** **6**.

**Table** **3** highlights ionic conductivity, activation energy, and electrochemical characteristics and classifies sulfide electrolytes according to their manufacturing techniques. Mechanochemical processes often produce high ionic conductivity and steady electrochemical performance when paired with heat treatment or liquid‐assisted techniques. This makes them appropriate for a variety of applications, including ASSBs. The commonly used process of ball milling frequently entails further annealing or sintering to improve conductivity and stability, as demonstrated in compositions such as Li_6.5_Sb_0.5_Ge_0.5_S_5_I and Li_6_PS_5_Cl. Liquid‐phase syntheses, including microwave‐assisted wet processes or dual solvent methods, provide faster reaction times and controlled particle morphology, improving interfacial stability and ionic transport. High‐temperature methods like melt quenching or thermal decomposition yield glass ceramics or metastable phases with exceptional ionic conductivities. Although coating strategies for electrolyte‐cathode interfaces, including polyphosphoric acid or alkoxide precursor, improve contact durability, their scalability and cost can vary. Takada et al. have made significant progress in interfacial engineering for SSBs by introducing a nanoscale Li_4_Ti_5_O_12_ layer between the oxide cathode and the sulfide electrolyte. This made the interface much less resistant and improved performance at high rates. This work paved the way for further research into enhancing interfaces. Interfacial modification remains crucial for maximizing the performance of sulfide‐based SSBs as coating technologies and material choices continue to improve.^[^ ^143^ ^]^ These methods of synthesis show how important processing is in designing sulfide electrolytes for advanced battery technology.

**Table 3:** Synthesis techniques and electrochemical performance of sulfide‐based solid electrolytes.

Sulfide electrolytes	Synthesis method	Ionic conductivity	Activation energy	Electrochemical properties	Voltage	Remarks	Refs.
0.37LiI·0.25Li_3_PS_4_·0.38Li_4_SnS_4_	Mechanochemical + heat treatment (200 °C)	5.5 × 10^−4^ S cm^−1^	28 kJ mol^−1^	Best rate performance in NMC+LiNbO_3_	2.4–3.6 V vs Li‐In	High conductivity, air stability similar to Li_4_SnS_4_	[130]
35LiI‐65(3Li_2_S‐1P_2_S_5_)	Liquid‐assisted mechanochemical synthesis	1.2 mS cm^−1^	33.3 kJ mol^−1^	Electrolytes produced using the liquid method achieve a critical current density of up to 0.8 mA cm^−2^	1.6–2.8 V vs Li/Li⁺	Improved Li interface stability due to reduced particle size	[131]
Na_3_SbS_4_	Ball milling + sintering at 250 °C, 12 h	3.10 × 10^−4^ S cm^−1^	0.21 eV	Discharge capacity 106 mAh g^−1^, Coulombic efficiency 99.3% (30 cycles)	2.0–3.8 V vs Na/Na⁺	High conductivity, optimal Ea, and stable electrochemical performance	[132]
Li_6.8_Si_0.8_As_0.2_S_5_I (LASI‐80Si)	Ball milling + annealing at 550 °C, 12 h	10.4 × 10^−3^ S cm^−1^	0.20 eV	Discharge capacity 216.9 mAh g^−1^, Coulombic efficiency 99.06% (up to 62 500 cycles)	0.9–2.4 V vs Li‐In/Li⁺	Superionic conductivity, high cycle life, and air stability, suitable for ASSBs	[133]
Li_5.5_PS_4.5_Cl_1.5_(Li_6_PS_5_Cl)	Ball milling + sintering	10.2 × 10^−3^ S cm^−1^	‐	1.95 mA cm^−2^ at 500 cycles	1.5–4.2 V vs Li⁺/Li	Moderate conductivity and oxidation stability	[133]
Na_3_SbS_4_	Aqueous solution route	0.1–0.2 mS cm^−1^ at 25 °C	0.27–0.35 eV	Discharge capacity of 256–346 mAh g^−1^, good reversibility (50 cycles)	0.6–3.0 V vs Na/Na⁺	Solution process minimizes toxic H_2_S evolution and enables uniform FeS_2_ coatings	[134]
Li_6_PS_5_Cl	Microwave‐assisted wet synthesis	2.79 mS cm^−1^	0.26 eV	Discharge capacity 145.5 mAh g^−1^ (0.5 C, 200 cycles, 0.12% capacity loss per cycle)	≈1.7 V	Short synthesis time, high Li‐ion conductivity, excellent stability	[135]
β‐Li_3_PS_4_	Wet‐chemical synthesis via THF‐mediated reaction at RT; heating at 140 °C to remove THF	1.6 × 10^−4^ S cm^−1^	0.356 eV	Broad electrochemical window (up to 5 V), stable against Li metal	0–5 V vs Li/Li⁺	Nanoporous structure enhances surface conduction, stabilizes metastable β phase, superior stability with Li	[84]
Li_7_P_3_S_11_	Liquid‐phase synthesis + heat treatment at 220 °C	1.0 × 10^−3^ S cm^−1^	12.8 kJ mol^−1^	Wide electrochemical stability window up to 5 V (vs. Li/Li⁺)	Up to 5 V vs Li/Li⁺	High ionic conductivity due to P_2_S_7_^4−^ units	[136]
β‐Li_3_PS_4_	Thermal decomposition of Li_3_PS_4_·ACN/Li_3_PS_4_·DME complexes	1.0 × 10^−4^ S cm^−1^ (ACN), 0.7 × 10^−4^ S cm^−1^ (DME)	26.8 kJ mol^−1^ (ACN), 30.5 kJ/mol (DME)	Good cycle performance in ASSBs with NMC cathode	–	High Li⁺ conductivity at RT, low Ea; compatible with Li‐metal anodes	[137]
Li_3.875_Sn_0.875_ As_0.125_S_4_	One‐step gas‐phase synthesis (500 °C)	2.45 × 10^−3^ S cm^−1^	0.273 eV	Discharge capacity 188.4 mAh/g, cycle life 210 cycles, 91.5% retention	0.6–3.2 V	Highest ionic conductivity, excellent air stability, and recoverability	[98]
70Li_2_S·30P_2_S_5_	Melt quenching at 750 °C	2.1 × 10^−3^ S cm^−1^	15 kJ mol^−1^	Li‐ion transport number close to 1	–	Superionic conductivity, ideal for ASSBs, with single‐phase Li_7_P_3_S_11_	[138]
Li_2_S‐P_2_S_5_ glass‐ceramic	Pelletizing at 94 MPa, heat treatment at 280 °C	1.7 × 10^−2^ S cm^−1^	17 kJ mol^−1^	High stability	0–5 V vs Li/Li⁺	Exceptional ionic conductivity exceeding that of liquid electrolytes, low grain boundary resistance after unification	[100]
Li_6.5_Sb_0.5_Ge_0.5_S_5_I	High‐energy ball milling + annealing at 450 °C	16.1 mS cm^−1^	0.18 eV	Discharge capacity 164 mAh g^−1^, good cycle stability	2.0–3.6 V vs Li/Li⁺	Very high conductivity, low Ea, improved air stability	[127]
70Li_2_S‐30P_2_S_5_	Ball milling + annealing at 210–250 °C	1.38 × 10^−3^ S cm^−1^	–	High reversible capacity (312 mAh g^−1^); 90.6% retention over 500 cycles	1.5–3.5 V vs Li/Li⁺	High cycle stability due to improved interfacial stability and the soft mechanical properties of PT and 70Li_2_S‐30P_2_S_5_	[139]
Li_6_PS_5_Cl	Ball milling	1.4 mS cm^−1^	–	Discharge capacity 600 mAh g^−1^, capacity retention 80.8% (500 cycles)	0.4–3 V vs In/LiIn	High energy density (1140 Wh kg^−1^), scalable dry‐film processing, stable cycling performance	[140]
Li_10_GeP_2_S_12_	EDA–EDT solution synthesis + heat treatment	0.74 mS cm^−1^	–	High reversible capacity and stable cycling	–	Good performance with low Ea, residual carbon present	[73]
Li_3‐x_PS_4_(x = 0.15)	Wet‐chemical with dual solvents (THF + o‐xylene)	0.2 mS cm^−1^ at 30 °C	–	Reversible capacity of 130 mAh g^−1^ in LiCoO_2_	3.0–4.3 V vs Li/Li⁺	Dual solvents yield narrow particle distribution, higher purity, and P_2_S_7_^4−^ phases; improved performance	[141]
Li_6_PS_5_Cl + Li_3_PO_4_ cathode coating	Polyphosphoric acid and Li acetate/Li ethoxide coating	≈10^−^⁷ S cm^−1^	–	Enhanced discharge capacity and stability at the cathode/electrolyte interface	2.5–4.25 V	Low‐cost, effective suppression of interfacial side reactions, suitable for mass production	[142]
Li_6_PS_5_Cl + Li_2_ZrO_3_	Alkoxide precursor coating	≈10^−6^ S cm^−1^	–	Moderate discharge capacity, stable interface	2.5–4.25 V	Chemically stable, but higher cost than Li_3_PO_4_	[142]

**Figure 7:**
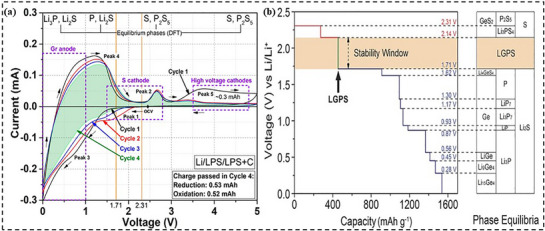
a) Cyclic voltammetry analysis of Li/LPS/WE cell. Adapted with permission.^[^ ^144^ ^]^ Copyright 2019, American Chemical Society; b) The results of first‐principles calculations on the voltage profile and phase equilibria of the LGPS sulfide solid electrolyte during lithiation and delithiation. Adapted with permission.^[^ ^154^ ^] Copyright 2016, Wiley‐VCH Verlag GmbH & Co. KGaA, Weinheim.^

Tushar et al.^[^ ^144^ ^]^ studied β‐Li_3_PS_4_ (LPS) and found its stability range (1.71–2.31 V by DFT) to be narrow. Experimentally, composite working electrodes (WE) contain ≈50 mg of C, LPS, (LPS+C), or (LPS+C+S/P) and notably, LPS decomposes outside and within this range. While oxidation (>2.31 V) forms insulating sulfur‐based layers, increasing cathode impedance irreversibly, reduction (<1.71 V) creates Li_2_S and Li_3_P, with partial reversibility and in turn lowers the anode impedance. These behaviors limit LPS performance in ASSBs. As shown in **Figure** **7**a, the cyclic voltammetry (CV) graph highlights the electrochemical behavior of LPS with carbon (LPS+C) over four cycles. The thermodynamic stability window of 1.71–2.31 V, marked in orange, shows decomposition reactions outside this range. On reduction, Peak 1 (1.71–2.31 V) corresponds to the formation of Li_2_S and Li_3_P, partially reversible, while Peak 3 (below 1.5 V) indicates further phosphorus reduction. On oxidation, Peak 5 (above 2.31 V) reflects the formation of S, P_2_S_5_, leading to a passivating layer that increases the resistance. In Cycle 4, the charge passed during reduction (0.53 mAh) and oxidation (0.52 mAh) processes are nearly equal, suggesting partial redox reversibility, though high‐voltage oxidation creates irreversible insulating products. Successive cycles stabilize redox currents slightly as peak intensities at high voltages decrease due to passivating layer formation. The CV results reinforce the need for strategies like voltage regulation,^[^ ^145^ ^]^ protective coatings materials like metal oxides (LiNbO_3_),^[^ ^146^, ^147^ ^]^ solid electrolyte interphase (SEI),^[^ ^148^ ^]^ and artificial SEI layers,^[^ ^149^ ^]^ use of composite electrodes,^[^ ^150^, ^151^ ^]^ and elemental additives as redox standards.^[^ ^152^, ^153^ ^]^

Han et al.^[^ ^154^ ^]^ investigated the electrochemical stability of SEs Li_10_GeP_2_S_12_and Li_7_La_3_Zr_2_O_12_ (LLZO), finding a significant discrepancy between their apparent and intrinsic stability windows using a novel Li/solid electrolyte/inert metal (Pt); Li/LGPS/LGPS‐C/Pt cell semi‐blocking electrode configuration was introduced, enhancing the contact and reaction kinetics via the use of carbon additives. First‐principles simulations for LGPS showed an intrinsic stability window of 1.7–2.1 V, which is significantly smaller than the 0–5 V previously claimed. This inherent stability window is depicted as a shaded area in Figure 7b, emphasizing the limited voltage range. While oxidation above 2.1 V forms Li_3_PS_4_, P_2_S_5_, GeS_2_, and S (red curve), LGPS lowers below 1.7 V to Li_2_S, Li_15_Ge_4,_ and Li_3_P, with separate reaction steps apparent as plateaus in the lithiation process (blue curve). Interphase development from these breakdown processes reduces ionic conductivity and raises interfacial resistance. This improved comprehension, as shown in Figure 7b, emphasizes the importance of the control voltage in order to stop degradation precisely and maximize SSB efficiency.

Voltage stability in SSBs depends on the solid electrolyte's chemical and electrochemical stability as well as its ionic conductivity, which collectively ensure resistance to degradation, compatibility with high‐voltage cathodes, and efficient charge transport. Oxygen doping in sulfide‐based electrolytes, such as Li_5.5_PS_4.425_O_0.075_Cl_1.5_, significantly enhances the performance by reducing the cathode reactivity, suppressing gas formation, improving moisture resistance, and enabling stable cycling at up to 4.5 V while mitigating H_2_S generation without compromising the ionic conductivity.^[^ ^155^ ^]^ Furthermore, a viable remedy for high‐voltage sulfide‐based SSBs is the use of small‐size single‐crystal cathodes with Li_2_O pre‐lithiation, which overcomes issues such as side reactions, low ionic conductivity, and volume variations during cycling.^[^ ^156^ ^]^ Superior performance and durability are ensured for high‐energy cathode materials like LCO by surface coatings with high Li⁺ conductivity materials like LLZO and LNO, which effectively inhibit interfacial side reactions, improve cycling stability, and retain capacity.^[^ ^157^ ^]^ A glassy/ceramic SEI stabilizes the interface between LCO and LGPS by suppressing parasitic reactions and blocking decomposition products, while its compact, boundary‐free coating enhances Li ion diffusion. The thermodynamic stability of the SEI, along with the high mutual reaction energies of components like Li_2_TiO_3_ and Li_x_B_y_O_z_, prevents interfacial degradation, and its mechanical robustness resists cracking and internal stress during charge/discharge cycles, ensuring long‐term performance.^[^ ^158^ ^]^

### 3.4 Mechanical Properties

The evaluation of materials used in ASSBs, particularly SEs, requires the consideration of mechanical properties. Hardness H, which represents resistance to surface deformation; fracture toughness K_Ic_, which indicates resistance to crack development; and Young's modulus E, which measures stiffness, are important factors. These characteristics determine the material's capacity to withstand strains and stresses during battery operation. Oxide‐based electrolytes such as garnet‐type Li_7_La_3_Zr_2_O_12_ typically have a high Young's modulus of 124–163 Gigapascal (GPa) and hardness values around 9–11.8 GPa,^[^ ^26^ ^]^ while polymer electrolytes such as PEO‐based systems exhibit much lower Young's modulus values (1–3 GPa) and hardness values (0.4‐0.7 GPa).^[^ ^9^ ^]^ Sulfide electrolytes generally fall between these extremes. For example, a Young's modulus of ≈18.5 GPa for Li_2_S‐P_2_S_5_ is considered low compared to oxide‐based electrolytes, enabling strain accommodation. In comparison, a hardness of ≈1.9 GPa is moderate between the soft polymer and rigid oxide systems. However, they are prone to breaking because of their poor fracture toughness (≈0.23 MPa·m^1/2^), which might impair battery performance.^[^ ^159^ ^]^ Developing long‐lasting and effective batteries requires an understanding of and attention to these aspects. A thorough examination of SEs based on sulfides provides significant new information on how they interact mechanically and electrochemically. Due to its Young's modulus, which ranges from 0.3 to 21.7 GPa, LGPS can deform under pressure (such as 100 MPa) to improve particle contact and decrease voids, both of which are essential for effective ion transport.^[^ ^160^ ^]^ Li_5.5_PS_4.5_Br_1.5_ has a Young's modulus of 25.3 GPa and Poisson's ratio of 0.35. Its ionic conductivity increases with pressure, saturating at 300 MPa as voids and grain boundaries are reduced, enabling efficient ion transport and processability.^[^ ^161^ ^]^ Due to higher Li_2_S concentration and packing density, the Young's modulus of Li_2_S‐P_2_S_5_ glassy SEs ranges from 14 to 25 GPa. Hot‐pressed pellets have higher values (18–25 GPa) than cold‐pressed ones (14–17 GPa). Because of the lower ion packing density and bond dissociation energy of sulfides, these moduli are smaller than those of oxide‐based electrolytes. High processability was indicated by the modest plastic deformation found in compression testing at 130 MPa. Because of these properties, Li_2_S‐P_2_S_5_ glasses combine mechanical flexibility with effective densification in moderate conditions, making them ideal for solid‐state batteries.^[^ ^162^ ^]^ The mechanical behavior of Li_2_S‐P_2_S_5_‐based glasses is dominated by visco‐plastic deformation. These glasses have a Young's modulus of around 10 GPa, a static yield strength of 0.2 GPa, and a high ionic conductivity of ≈7 × 10^−4^ Scm^−1^ at ambient temperature. By allowing conformal contact with Li metal electrodes at moderate pressures, this viscoplasticity inhibits the production of dendrites. Nanoindentation tests and finite element modelling (FEM) verify that viscoplastic creep, not viscoelasticity, controls deformation, lowering stress concentrations at crack tips, lowering the probability of fracture, and enhancing resistance to Li dendritic penetration. By avoiding mechanical failure under high current densities, these properties improve the electrolyte's durability and provide fast‐charging capabilities.^[^ ^163^ ^]^ The mechanical properties of sulfide electrolytes, including moderate Young's modulus, low hardness, and limited fracture toughness, enable strain accommodation, reduce voids, and improve ion transport under pressure. Viscoplastic creep behavior inhibits cracking and dendrite formation, ensuring durability and processability in SSBs.

### 3.5 Scaleup Technology

Both potential and obstacles arise when sulfide‐based SSEs are produced on an industrial scale from laboratory research. Sulfide materials require careful handling and inert atmosphere processing because, in contrast to oxide SSEs, they are extremely sensitive to air and moisture, which increases the danger of deterioration and harmful H_2_S emissions. Achieving high density, low interfacial impedance, minimal defects, and economical scalability are important goals. While cutting‐edge processes like aerosol deposition and additive manufacturing (AM) provide solvent‐free substitutes for materials that are sensitive to moisture, more traditional techniques like tape casting, screen printing, and electrophoretic deposition (EPD) have been modified for the creation of thin films with no defects. Sintering techniques such as ultrafast high‐temperature sintering (UHS) and spark plasma sintering (SPS), that balance cost, scalability, and efficiency, are being investigated for densification in order to improve material performance and throughput. **Table** **4** explains some of these technologies.^[^ ^164^ ^]^

**Table 4:** Overview of sulfide Electrolyte technologies: thickness, advantages, challenges, and prospects.

Sulfide electrolyte technology	Thickness range [µm]	Advantages	Challenges	Prospects	Refs.
Tape casting	8–70	Large‐scale production; uniform thickness, Proven ionic conductivity (≈1–2 mS cm^−1^)	Limited solvent options; sulfide decomposition in polar solvents	Promising commercial scaling with optimized binder‐solvent systems	[165]
Composite electrolytes (Li_6_PS_5_Cl/PVDF)	100–120	Improved cycling stability against Li, processable into free‐standing membranes.	Reduced ionic conductivity at higher polymer content; balancing percolation paths remains a challenge.	Promising for enhancing long‐term safety and stability in ASSBs	[166]
Hot‐pressed, aramid‐fiber‐reinforced (Li_2_S)70(P_2_S_5_)30	100	High ionic conductivity (up to 2.4 mS cm^−1^), flexible, >98% dense, improved mechanical robustness	Microcracking under compressive stress, sensitivity to moisture, and potential residual thermal stresses	LiI doping, compliant interlayers to reduce stack pressure	[167]
Wet coating (Li_9.88_GeP_1.96_Sb_0.04_S_11.88_Cl_0.12_)	8–50	Low‐cost, scalable, adaptable to various substrates, and ultrathin achievable.	Requires solvent‐stable compositions, solvent interaction can affect performance.	Promising for high‐energy‐density batteries, especially with advanced doped compositions.	[168]
Electrospinning of polyimide (PI) nanowoven scaffolds: Li_6_PS_5_Cl_0.5_Br_0.5_	40–70	Thin SE layers improve cell energy density, ionic conductivity 2.0 mS cm^−1^, Stable up to 400 °C.	Developing electrodes that withstand similar heat treatment conditions.	Improve SE composition and optimize halide doping.	[169]
Self‐limited assembly strategy/blade‐coating: Li_6_PS_5_Cl	60	Ultralight, flexible, high ionic conductivity (6.3 mS cm^−1^), improved mechanical properties	Requires optimization for active material loading and interfacial stability	Promising for high‐energy‐density applications and flexible devices	[170]
Ethyl cellulose‐based SE membranes: Li_6_PS_5_Cl	47	Ultrathin, lightweight, robust, excellent ionic conductivity (1.65 mS cm^−1^), low areal resistance (4.32 Ω·cm^2^), scalable	Challenges in maintaining flexibility and strength with reduced thickness	Promising for achieving high‐energy‐density batteries, suitable for industrial‐scale manufacturing	[171]
Dry synthesis: LGPS/PTFE	100	Solvent‐free process, high ionic conductivity (3.6 × 10^−4^ S cm^−1^), scalable production, low cost	Slight decrease in conductivity, interface challenges with electrodes	Roll‐to‐roll manufacturing for industrial scalability, enhanced interface engineering	[172]
Hybrid sulfide/polymer: LGPS/PEO/CTMS/NM	60	High ionic conductivity, wide electrochemical stability, excellent mechanical strength, nonflammability, and high compatibility with Li anodes.	Mitigating parasitic reactions with Li metal, achieving large‐scale fabrication while maintaining performance.	Roll‐to‐roll scalable manufacturing, practical use in high‐safety, high‐energy, and potential applications in flexible electronics.	[173]

Although they need more work, solvent‐free techniques like hot pressing and dry synthesis show promise. Enhancing interfacial stability, establishing roll‐to‐roll manufacturing for scalability, and optimizing binder‐solvent systems are the main focuses of industrial strategies. New AM methods with the ability to create strong, ultrathin SSE layers include hybrid composites and stereolithography. To overcome material problems, lower costs, and facilitate the wider deployment of SSBs, cooperation between academia and industry is essential.

### 3.6 Safety

Production settings need ongoing H_2_S monitoring, worker safety gear, and maybe odor‐mitigation techniques to guarantee worker safety. Stricter controls and localized safety measures are essential for occupational health compliance, even if the present dry room regulations for LIBs production can be modified for sulfide‐based ASSBs.^[^ ^174^, ^175^ ^]^ To prevent H_2_S formation and material degradation, sulfide‐based material handling requires dry room conditions with dew points between −40 and −50 °C. High humidity leads to cracking and deformation of electrolytes, while no H_2_S is detected below −50 °C using dry air or argon atmospheres. Limiting exposed sulfide surfaces and maintaining H_2_S concentrations below 5 ppm ensures worker safety.^[^ ^168^ ^]^ As shown in **Table** **5**, sulfide electrolytes perform better than liquid electrolytes in terms of environmental, electrical, and thermal safety in ASSBs. Advanced battery applications are safer and more stable due to their delayed heat release, resistance to shorting, ability to withstand higher voltages with little capacity loss, and self‐shutdown when exposed to air.^[^ ^176^ ^]^

**Table 5:** Comparison of sulfide electrolytes for ASSBs and liquid electrolytes for LIBs.

Aspect	Sulfide electrolytes ASSBs	Liquid electrolytes LIBs	Refs.
Thermal safety	Gradual heat release, delayed peak at 372 °C, low enthalpy (114.6 J g^−1^).	Sharp heat release, peaks at 230 °C and 265 °C, high enthalpy (300 J/g).	[176, 177, 178]
State of charge	High SoC: Gradual thermal activity prevents runaway.	High SoC: Risk of localized heating and runaway.	[176]
Short‐circuit resistance	Maintains stability up to 160 °C, resists shorting. High temperatures accelerate degradation, causing a marginal voltage drop in ASSBs	Separator shrinkage at ≈160 °C causes voltage drops.	[176, 179, 180]
Overcharge stability	stable voltage profiles and minimal capacity loss, handling high‐voltage stress while being charged to 5.5 V	Liquide electrolyte: suffers irreversible structural damage, microshorts, and rapid capacity decay when overcharged	[176, 181]
Air exposure	H_2_S levels detected were <25 ppm, causing fatigue, headaches, and dizziness, but not life‐threatening effects	LIBs can release various lethal gases, including hydrogen fluoride (HF)	[182, 183]
Environmental impact	LPSCl is more stable, with minimal gas release compared to LPSBI.	Higher degradation and environmental impact under air exposure.	[184, 185]

The sulfide solid electrolyte is a viable option for safer and more effective ASSBs because of its high ionic conductivity, non‐flammability, and suitability for low‐pressure operation and cycle stability.^[^ ^186^ ^]^

### 3.7 Advantages and Challenges

Sulfide electrolytes have shown great promise in the development of SSBs and provide several benefits. Sulfide electrolytes are notable for their exceptionally i) high ionic conductivity, often rivalling or exceeding that of liquid electrolytes, with many achieving conductivities above 10 mS cm^−1^.^[^ ^165^ ^]^ For example, Li_9.54_[Si_0.6_Ge_0.4_]_1.74_P_1.44_S_11.1_Br_0.3_O_0.6_ demonstrates an impressive bulk ionic conductivity of 32 mS cm^−1^ at RT,^[^ ^187^ ^]^ essential for efficient Li‐ion transport, supporting batteries with high power and energy densities;^[^ ^108^ ^]^ ii) They greatly enhance the safety of SSBs by being solid and non‐flammable, unlike conventional liquid electrolytes that are flammable and prone to leakage, significantly lowering the risk of thermal runaway;^[^ ^188^ ^]^ iii) They exhibit favorable mechanical properties that enhance solid‐state battery design, enabling excellent contact at solid‐solid interfaces between the electrolyte and electrodes;^[^ ^58^, ^189^ ^]^ iv) They are adaptable for applications with different voltage needs because of their broad electrochemical window, which allows them to operate across a wide voltage range without decomposing and supports higher energy densities;^[^ ^190^ ^]^ v) They exhibit excellent compatibility with Li metal anodes by resolving problems like dendritic formation that are common in liquid electrolyte systems, and facilitating the use of these anodes, known for their high theoretical capacity.^[^ ^102^, ^191^ ^]^ vi) They use comparatively abundant raw materials, which could reduce production costs and increase economic feasibility in large‐scale applications. As manufacturing techniques develop and expand, cost‐effectiveness should continue to improve.^[^ ^192^ ^]^

**Figure 8:**
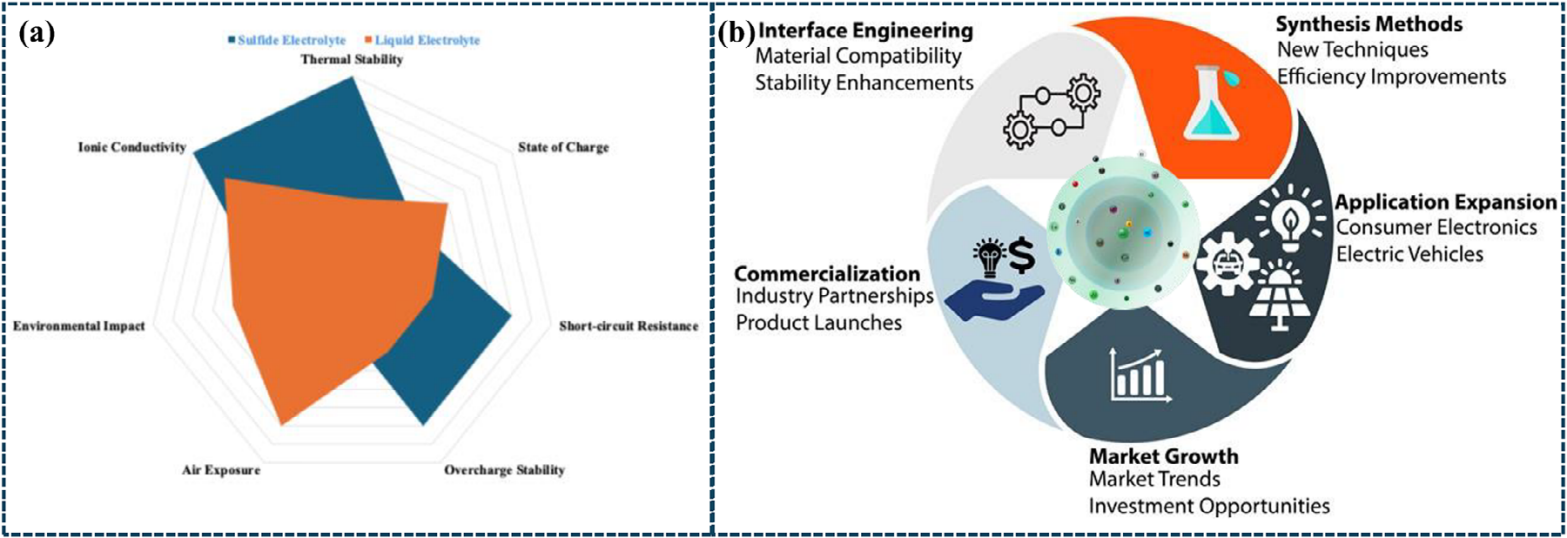
a) Comparative performance of sulfide vs liquid electrolytes in lithium battery technology, b) Key focus areas for advancing sulfide electrolyte technologies in solid‐state batteries.

Although sulfide electrolytes have many advantages, there are also key weaknesses that need to be overcome for SSBs to realize their potential for widespread market adoption. However, these electrolytes are chemically unstable to moisture, releasing toxic H_2_S gas. Therefore, their manufacturing requires stringent controls, and devices must be coated to protect from contact with air.^[^ ^193^ ^]^ Interfacial instability between sulfide‐based electrolytes and electrode materials also lowers the efficiency of the system. It raises the resistance by creating layers that make it harder for Li ions to move.^[^ ^194^, ^195^ ^]^ Their brittle nature poses challenges for applications requiring high energy density, flexibility, or structural integrity, as they are prone to breaking under mechanical stress or during cycling.^[^ ^196^ ^]^ Additionally, the need for complex and expensive production systems for high‐purity sulfide materials hampers the cost‐effectiveness of these batteries, and the challenge of scalability without compromising performance and quality remains significant.^[^ ^197^ ^]^ An additional concern is that sulfide electrolytes promote dendritic growth on Li metal anodes. This can break the electrolyte and lead to short circuits, so effective suppression techniques are needed to make sure the system works safely and reliably.^[^ ^198^, ^199^ ^]^ **Figure** **8**a.

Sulfide electrolytes in SSBs have a bright future due to continuous research and development activities aimed at removing present constraints. Sulfide electrolytes have the potential to revolutionize industries as they play a significant part in the next generation of safe, affordable, and high‐performance energy storage systems.^[^ ^200^ ^]^ Figure 8b.

## 4 Halide Electrolytes

Halide SSEs are a family of ionic conductors where Li‐ion transport is facilitated by the halide anions (F^−^, Cl^−^, Br^−^, and I^−^). These compounds are made up of different cations, which may be metals or non‐metals, halide anions, and Li. The distinct chemical and structural characteristics make it possible to use them in ASSBs. When Li‐ion conductive materials like LiX (X = F, Cl, Br, I) were discovered in the 1930s,^[^ ^201^ ^]^ it was discovered that LiI had a comparatively low ionic conductivity of 10^−7^ S cm^−1^,^[^ ^202^ ^]^ marking the beginning of the history of halide‐based electrolytes.^[^ ^203^ ^]^ The 1970s witnessed tremendous progress, most notably the development of LiAlCl_4_ in 1976, which at RT had a conductivity of 1.2 × 10^−6^ S cm^−1^.^[^ ^204^ ^]^ An important turning point in solid‐state battery technology was reached in 1969 with the introduction of the first all‐solid‐state LiI/AgI battery.^[^ ^205^ ^]^ In the 1980s, Ryoji Kanno et al. demonstrated improvements with Li_2_CrCl_4_ (6.3 × 10^−2^ S cm^−1^ at 400 °C,^[^ ^206^ ^]^ Li_2_CoCl_4_ 5.0× 10^−2^ at 400 °C,^[^ ^207^ ^]^ and Li_1.6_Mg_l.2_CI_4_ at RT.^[^ ^208^ ^]^ Furthermore, H. D. Lutz et al. discovered that halide spinels Li_2_MCl_4_ with M = Mg, Mn, Fe, and Cd showed very high Li ionic conductivity.^[^ ^209^ ^]^ The 1990s saw significant advancement with Li_3_InCl_6_ reaching 0.2 S cm^−1^ at 300 °C^[^ ^210^ ^]^ and Andreas Bohnsack et al. exploring mixed halides such as Li_3_MCI_6_ with (M = Tb‐Lu, Y, Sc),^[^ ^211^ ^]^ and Li_3_MBr with (M = Sm‐Lu, Y), which also showed notable conductivity improvements.^[^ ^212^ ^]^ In the 2000s, Tomita et al. focused on high‐temperature phases like Li_3_InBr_6,_^[^ ^213^ ^]^ and LiInBr_4,_^[^ ^214^ ^]^ achieving 10^−3^ S cm^−1^, while Li_3_InBr_3_Cl_3_ reached 1.2 × 10^−4^ S cm^−1.[^ ^215^ ^]^ The 2010s marked modern advancements, with the development of a family of anti‐perovskites having high ionic conductivities, such as Li_3_OCl, Li_3_OBr, and their mixed compounds like Li_3_OCl_0.5_Br_0.5_, a promising area of research for SEs.^[^ ^216^, ^217^ ^]^ The development of solid halide electrolytes during the last ten years will be addressed in this section.

### 4.1 Composition and Structure

Owing to the special chemical characteristics of halogen anions, halide SSEs are seen as the most promising options for developing ASSBs technology.^[^ ^40^ ^]^ Halide SSEs can be classified into four main categories based on their composition and structural features,^[^ ^203^ ^]^ as follows: The first category includes **halide SSEs with group 3 elements (Sc, Y, La–Lu)**, commonly represented by the formula Li‐M‐X (M = Sc, Y, La‐Lu; X = F, Cl, Br, I). Examples include fluoride‐based compounds such as LiScF_4,_^[^ ^218^ ^]^ LiYF_4,_^[^ ^219^ ^]^ LiCeF_5,_^[^ ^220^ ^]^ LiYbF_4,_^[^ ^221^ ^]^ and LiLuF_4_;^[^ ^222^ ^]^ chloride‐based compounds like Li_3_ScCl_6,_^[^ ^223^ ^]^ Li_3_YCl_6,_^[^ ^224^ ^]^ Li_3_YbCl_6_, Li_3_LuCl_6,_^[^ ^211^ ^]^ and Li_2_ZrCl_6_;^[^ ^225^ ^]^ bromide‐based compounds including Li_3_ScBr_6,_^[^ ^223^ ^]^ Li_3_YBr_6,_^[^ ^223^ ^]^ Li_3_YbBr_6_, Li_3_LuBr_6_;^[^ ^203^ ^]^ and iodide‐based compounds such as LiScI_3_^[^ ^226^ ^]^ and Li_3_ErI_6._^[^ ^227^ ^]^These compounds exhibit structures such as orthorhombic, trigonal, and monoclinic and are characterized by high ionic conductivity at RT (up to 10^−3^ S.cm^−1^) due to intrinsic vacancies^[^ ^228^, ^229^ ^]^ The second category involves **halide SSEs with Group 13 elements (Al, Ga, In)**, typically described by the formula Li_3_MX_6_ (M = Al, Ga, In; X = F, Cl, Br, I). Examples based on the comparatively reduced ionic radius of Ga^3+^, Al^3+^, and In^3+^, such as LiGaCl_4,_^[^ ^230^ ^]^ LiAlCl_4,_^[^ ^231^ ^]^ Li_3_InCl_6,_^[^ ^232^ ^]^ Li_3_GaF_6,_^[^ ^233^ ^]^ and LiInI_4._^[^ ^234^ ^]^ The different coordination structures depend on the radius of cations and anions. These materials often have monoclinic or orthorhombic structures and moderate ionic conductivity (ranging from 10^−6^ to 10^−3^ S.cm^−1^). The third category encompasses **halide SSEs with divalent metal elements (Ti, V, Cr, Mn, Fe, Co, Ni, Cu, Zn, Cd, Mg, Pb)**, represented by formulas such as Li_2_MX_4_ and Li_6_MX_8_. These SSEs exhibit structural diversity, including olivine (Li_2_ZnI_4_),^[^ ^235^ ^]^ spinel (normal (Li_2_ZnCl_4_),^[^ ^236^ ^]^ inverse (Li_2_MnCl_4_),^[^ ^237^ ^]^ or deficient (Li_1.6_Mn_1.2_Cl_4_),^[^ ^238^ ^]^ and Suzuki phases (Li_6_FeCl_8_).^[^ ^239^ ^]^ They achieve improved ionic conductivity (up to 10^−2^ S.cm^−1^) via inverse spinel or deficient spinel structures. The fourth category includes **halide SSEs with non‐metal elements (N, O, S)**, generally formulated as LiMX (M = N, O, S; X = Cl, Br, I), which are further classified into three subgroups based on their composition: i) Lithium‐Nitrogen‐Halide (Li‐N‐X) SSEs include Li_3_NX (X = Cl, Br, I), such as Li_5_NI_2_;^[^ ^240^ ^]^ ii) Lithium‐Oxide‐Halide (Li‐O‐X) SSEs include Li_3_OCl and Li_3_OBr, which belong to Li‐rich anti‐perovskites,^[^ ^216^ ^]^ as well as lithium‐hydroxide halides (Li_3‐x_OH_x_Cl), such as Li_2_OHCl, which adopt anti‐perovskite structures;^[^ ^241^ ^]^ iii) Lithium‐Sulfur‐Halide (Li‐S‐X) SSEs include Li_3_SX (X = Cl, Br, I), such as Li_3_SBF_4_, which exhibits an anti‐perovskite structure.^[^ ^242^ ^]^ Additionally, a new phase with a double anti‐perovskite‐like structure, Li_6_OSI_2_, has also been reported.^[^ ^243^ ^]^ Existing halide Li_a_MX_β_ (M = metal element, X = F, Cl, Br, I) SSEs can be categorized based on the central metal cation M, including trivalent metal elements such as Sc, Y, and the lanthanides (La‐Lu), post‐transition metals like Al, Ga, and In, and divalent metal elements. Many halide‐based electrolytes with various close‐packed structures have been developed by mixing multivalent metal cations with halide anions of different electronegativities.^[^ ^244^ ^]^

**Figure 9:**
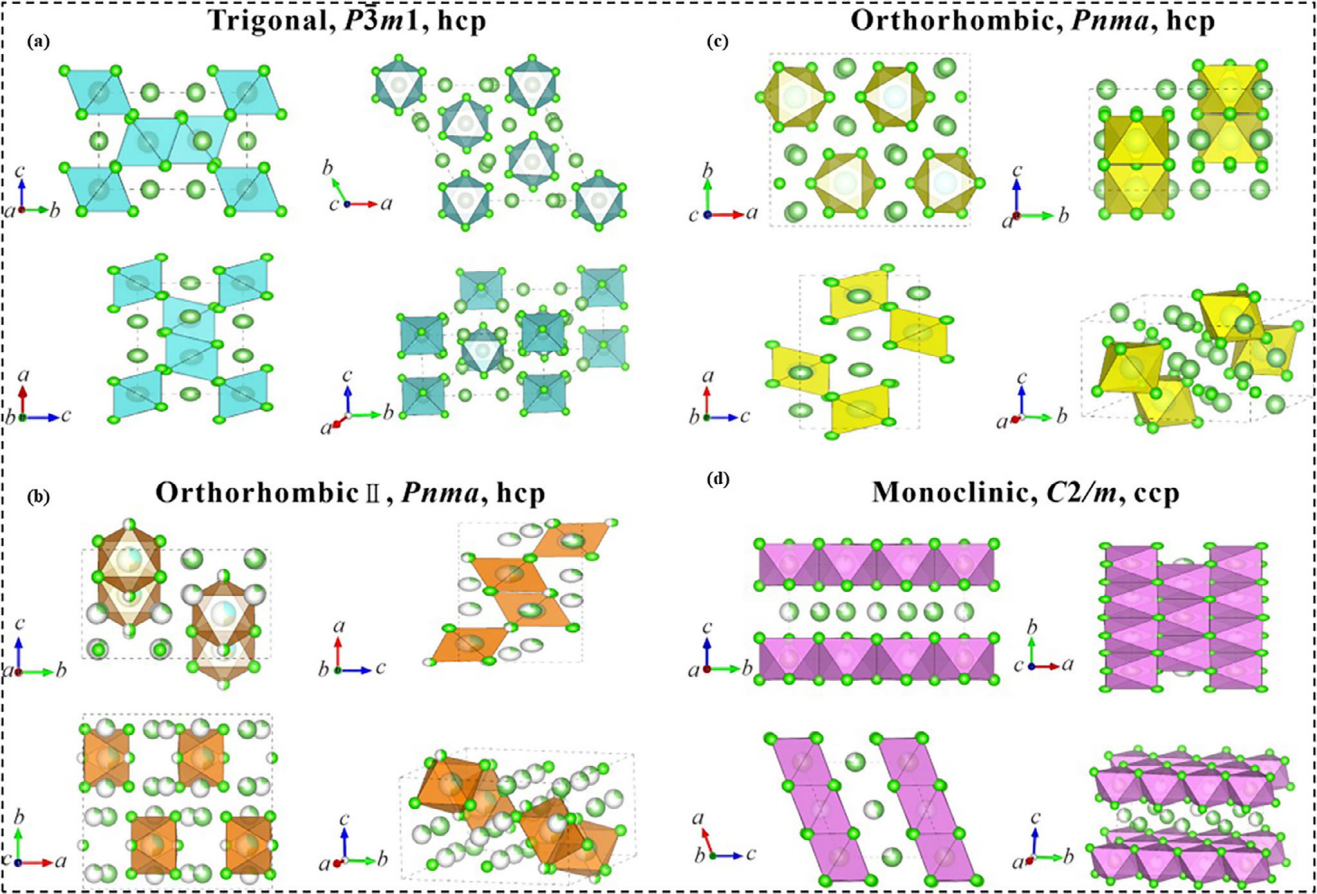
Outlined unit cells of halide solid electrolytes are shown for different crystal structures: a) trigonal, b) orthorhombic type I, c) orthorhombic type II, and d) monoclinic. Adapted with permission.^[^ ^244^ ^] Copyright 2023, Elsevier and Science Press.^

Li_3_MCl_6_ (M = Y, Yb, Lu, and Er) is an example of a trigonal (P3̅m1) structure that arises from a hexagonal close‐packed (hcp) anion arrangement,^[^ ^211^, ^224^, ^245^ ^]^ as seen in **Figure** **9**a. The orthorhombic structures, such as Li_3_YCl_6_^[^ ^210^ ^]^ and Li_3_YbCl_6_^[^ ^246^ ^]^ (space group Pnma), are derived from a hcp, as shown in Figure 9b,c. Similarly, other orthorhombic structures include Li_3_AlF_6_ (space group Pna2_1_)^[^ ^247^ ^]^ and compounds like Li_2_CoCl_4_^[^ ^207^ ^]^ and Li_2_FeCl_4,_^[^ ^248^ ^]^ which adopt an Imma space group. The monoclinic (C2/m) structure, found in compounds such as Li_3_MBr_6_ (M = Sm‐Lu, Y)^[^ ^212^ ^]^ and Li_3_ScCl_6,_^[^ ^211^ ^]^ is based on a cubic close‐packed (ccp) anion arrangement as shown in Figure 9d. Additionally, other monoclinic structures include Li_3_ErI_6_ (space group C2/c),^[^ ^227^ ^]^ Li_3_LaI_6_ (space group C2),^[^ ^249^ ^]^ LiInI_4_ (space group P2_1_/c),^[^ ^250^ ^]^ and LiGaBr_4_ (space group P2_1_/a).^[^ ^251^ ^]^

**Figure 10:**
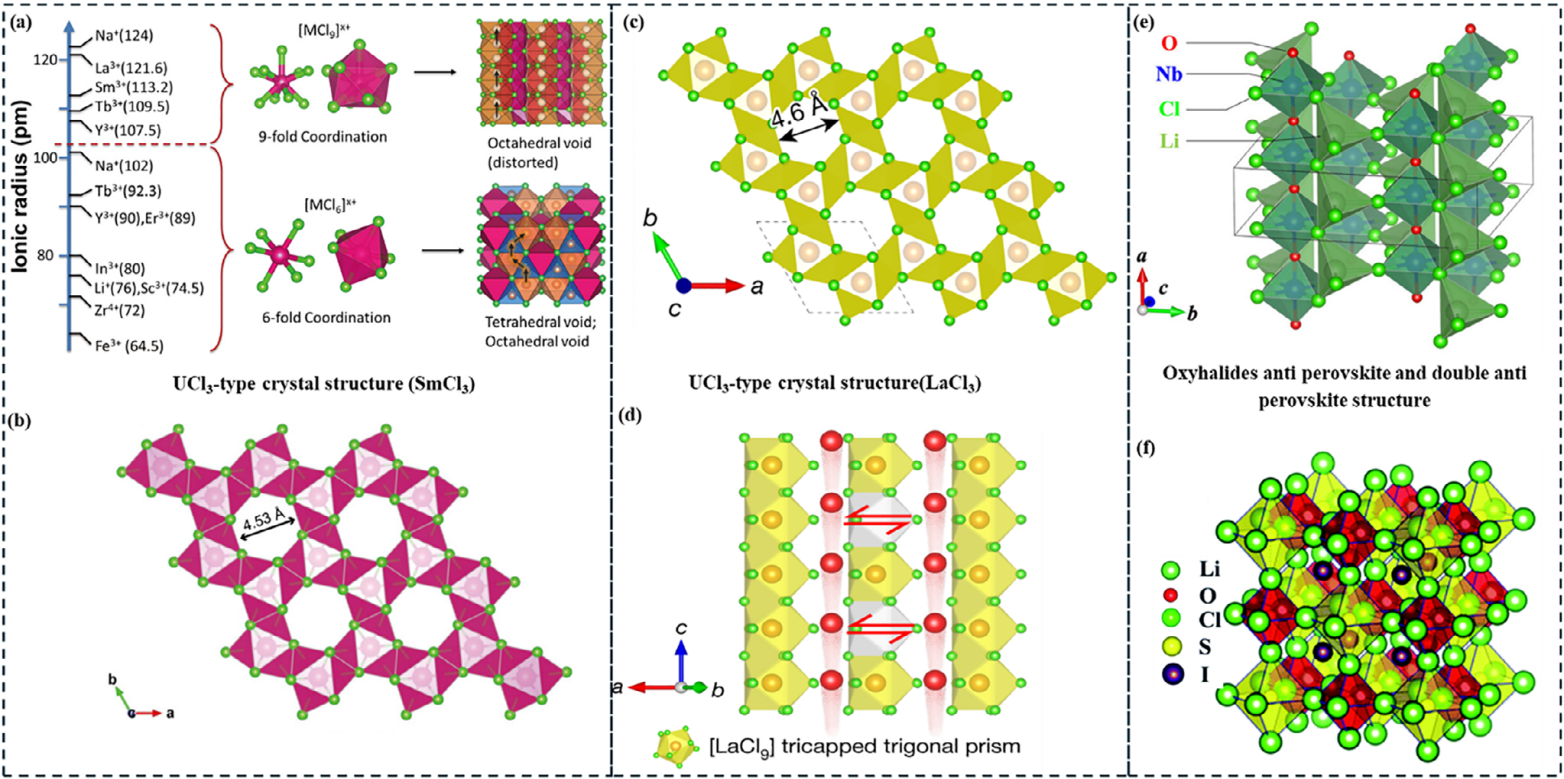
Various structures based on halides: a) The relationship between the ionic radius of M and the coordination of [MCl_x_] polyhedra. The black arrows on the crystal structures indicate the Li⁺ diffusion pathway. Adapted with permission.^[^ ^256^ ^]^ Copyright 2022, American Chemical Society; b) Top view of the SmCl_3_ lattice along the axis, highlighting the abundant channels with an inner diameter of 4.53 Å. Adapted with permission.^[^ ^256^ ^]^ Copyright 2022, American Chemical Society; c) Top view of the LaCl_3_ lattice along the c‐axis, illustrating the unit cell (dashed square) and the abundant, intrinsically pre‐existing channels of inner diameter 4.6 Å. Adapted with permission.^[^ ^263^ ^]^ Copyright 2023, Springer Nature Limited; d) Side view of the vacancy‐contained LaCl_3_ lattice indicating Li^+^ migration along the 1D channel (red spheres) and between adjacent channels (bidirectional arrows; vacancies are represented by the grey tricapped trigonal prisms). Adapted with permission.^[^ ^263^ ^]^ Copyright 2023, Springer Nature Limited; e)oxyhalide crystal structure of the LNOC. Adapted with permission.^[^ ^264^ ^]^ Copyright 2023, Wiley‐VCH GmbH; f) double anti‐perovskite structure, which is constructed using alternating Li_6_O and Li_6_S octahedrons over a face‐centered cubic lattice (Li_6_OSI_2_). Adapted with permission.^[^ ^243^ ^] Copyright 2018, Royal Society of Chemistry.^

As depicted in **Figure** **10**, structural disorder and amorphous content further enhance ion mobility, whereas the UCl_3_‐type hexagonal crystal structure offers an effective ion‐conducting framework.^[^ ^252^, ^253^ ^]^ This material, which was among the first chloride‐based universal cation conductors, is a promising option for sophisticated ion‐conducting applications that rely on materials such as LaCl_3_, CeCl_3_, and SmCl_3._^[^ ^254^, ^255^, ^256^ ^]^

Halide‐based electrolytes in ASSBs exhibit tunable Li diffusion properties depending on their structure, composition, anionic ratio, and the ionic radius of the metal cation (Figure 10a). Open‐framework halides such as LaCl_3_ and SmCl_3_ offer intrinsic channels for fast Li ion transport (Figure 10b–d). Additionally, defect engineering and mixed‐anion approaches, including oxyhalides anti‐perovskites (Figure 10e) and oxyhalides double anti‐perovskites (Figure 10f), further optimize the Li‐ion conductivity and electrochemical performance. Antiperovskite‐structured Li‐oxyhalide electrolytes (X_3_BA) are electrically inverted derivatives of traditional perovskites (ABX_3_),^[^ ^257^ ^]^ maintaining the same perovskite topology and 113 stoichiometry, however, in a reversed arrangement. A typical example is Li_3_OBr, where X is a monovalent cation (Li^+^), B is a divalent anion (O^2‐^), and A is a monovalent halide anion (Cl^−^ or Br^−^).^[^ ^258^, ^259^ ^]^ Li‐Ta(Nb, Hf, Zr)‐O‐Cl oxyhalide electrolytes represent a new frontier in SSB materials, offering a balance between high ionic conductivity, stability, and compatibility with advanced cathode materials.^[^ ^260^, ^261^, ^262^ ^]^

### 4.2 Ionic Conductivity

Owing to its small size, Li ion favors tetrahedral or octahedral coordination in solid‐state structures, where connected polyhedra form transport channels that directly influence ionic conductivity in Li‐based SEs.^[^ ^265^ ^]^ To develop high‐performance halide SEs (>1 mS cm^−1^) for ASSBs, key strategies include inducing a trigonal‐to‐monoclinic transition (Li_2_ZrCl_6_ → Li_3.1_ZrCl_4.9_O_1.1_), stabilizing Li^+^ interstitial sites via O^2‐^ substitution, and enhancing Li^+^ site disorder through aliovalent cation doping (Y^3+^, Sc^3+^, In^3+^). BVSE calculations optimize migration pathways and lower energy barriers, while nanostructuring and controlled sintering reduce the grain boundary resistance. Finally, optimizing electrode‐electrolyte interfaces ensures efficient Li ion transfer, collectively enabling next‐generation halide SEs with superior conductivity and stability.^[^ ^266^ ^]^ A comprehensive strategy focusing on crystalline structure and composition adjustments is essential for enhancing the ionic conductivity and stability of halide‐based SEs in ASSBs, as shown in **Table** **6**.

**Table 6:** The structures and conductivities of halide electrolytes.

Group	Halide materials	Structure	Ionic conductivity	Activation energy	Ref
SSEs with group 3 elements (Sc, Y, La‐Lu)	Li_3_YCl_6_	Trigonal P3̅m1	0.51 mS cm^−1^ @ RT	0.40 eV	[224]
SSEs with group 3 elements (Sc, Y, La‐Lu)	Li_3_YBr_6_	Monoclinic (C2/m)	1.72 mS cm^−1^ @ RT	0.37 eV	[224]
SSEs with group 13 elements (Al, Ga, In)	Li_3_InCl6	Monoclinic (C2/m)	1.49 mScm^−1^ @ RT	–	[267]
SSEs with group 13 elements (Al, Ga, In)	Li_3_InCl_6_	Monoclinic (C2/m)	2.04 mS cm^−1^ @ RT	0.347 eV	[268]
SSEs with group 3 elements (Sc, Y, La‐Lu)	Li_3_ScCl_6_	Monoclinic (C2/m)	3.02 mS cm^−1^ @ RT	0.25 ± 0.04 eV	[269]
SSEs with group 3 elements (Sc, **Y**, La‐Lu) and group 13 elements (Al, Ga, **In**)	Li_3_Y_1‐x_In_x_Cl_6_	hcp → ccp (anion sublattice transition)	1.09 to 1.42 mS cm^−1^ all samples with x ≥ 0.5	Varies with x (0 ≤ x <1)	[270]
SSEs with group 3 elements (Sc, Y, La‐Lu)	Li_3_YCl_6_	Trigonal P3̅m1	0.345 mS cm^−1^ @ RT	0.39 eV	[271]
UCl_3_‐type	BM‐SmCl_3_·0.5LiCl	Zeolite‐like (P6_3_/m, [SmCl_9_]^6−^ prisms) + LiCl (Fm‐3m)	0.12 mS cm^−1^ @30 °C	0.063 eV	[256]
UCl_3_‐type	BM‐SmCl_3_·0.5Li_2_ZrCl_6_	Zeolite‐like (P6_3_/m, [SmCl_9_] ^6−^ prisms) + Li_2_ZrCl_6_	1 mS cm^−1^ (@30 °C)	–	[256]
Fe^3^⁺‐substituted tetravalent metal‐based	Li_2.25_Zr_0.75_Fe_0.25_Cl_6_	hcp with Fe^3^⁺ substitution	0.98 mS cm^−1^ @30 °C	0.346 eV	[225]
Oxyhalide oxygen‐substituted	Li_3.1_ZrCl_4.9_O_1.1_	Monoclinic ccp	1.3 mS cm^−1^	Lower than Li_2_ZrCl_6_	[266]
SSEs with group 13 elements (Al, Ga, In)	Li_2.3_Hf_0.7_In_0.3_Cl_6_	Monoclinic	1.05 mS cm^−1^ @30 °C	0.337 eV	[244]
Group 3 elements (Sc, Y, La‐Lu)	Li _2.5_Y_0.5_Zr_0.5_Cl_6_	Trigonal	1.4 mS cm^−1^ @RT	0.33 eV	[272]
Group 3 elements (Sc, Y, La‐Lu)	Li_2.5_Er_0.633_Zr_0.367_Cl_6_	Trigonal	1.1 mS cm^−1^ @RT	0.34 eV	[272]
Group 3 elements (Sc, Y, La‐Lu)	Li_2_Sc_2/3_Cl_4_	Disordered spinel	1.50 mS cm^−1^ @30 °C	0.34 eV	[273]
Group 13 elements (Al, Ga, In)	Li_2.9_In_0.9_Zr_0.1_Cl_6_	Monoclinic	1.54 mS cm^−1^ @20 °C	0.296 eV	[274]
Group 13 elements (Al, Ga, In)	Li _2_In_0.444_Sc_0.222_Cl_4_	Monoclinic	2.03 mS cm^−1^ @RT	0.33 eV	[275]
Group 3 elements (Sc, Y, La‐Lu)	Li_2.556_Yb_0.492_Zr_0.492_Cl_6_	Orthogonal	1.58 mS cm^−1^ @RT	0.317 eV	[276]
Group 3 elements (Sc, Y, La‐Lu)	Li_3_Y(Br_3_Cl_3_)	Octahedral	7.2 mS cm^−1^ @RT	0.25 eV	[277]
Group 3 elements (Sc, Y, La‐Lu)	Li_2.375_Sc_0.375_Zr_0.625_Cl_6_	Monoclinic (C2/m)	2.2 mS cm^−1^ @RT	0.31 eV	[278]
Group 3 elements (Sc, Y, La‐Lu)	Li_2.4_Y_0.4_Zr_0.6_Cl_5.85_F_0.15_ Li_2.4_Y_0.4_Zr_0.6_Cl_6_	Trigonal Trigonal	1.45 mS cm^−1^ @RT 2.05 mS cm^−1^ @RT	0.30 eV 0.31 eV	[279]
Oxyhalide	LiNbOCl_4_ LiTaOCl_4_	Orthorhombic Orthorhombic	10.4 mS cm^−1^ @ RT 12.4 mS cm^−1^ @ RT	0.24 eV 0.23 eV	[264]
Oxyhalide (LaCl_3_‐based oxychloride)	Li_0.8_Zr_0.25_La_0.5_Cl_2.7_O_0.3_	Hexagonal	0.75 mS cm^−1^ @ RT	0.23 eV	[280]
Multiple‐cation mixed chloride in the UCl3 skeleton	Li−LaCeZrHfTa−Cl	Hexagonal	1.8 mS cm^−1^ @ RT	0.264–0.283 eV	[254]
High‐entropy halide SE with group 3 elements (Sc, Y, La‐Lu)	Li_2.75_Y_0.16_Er_0.16_Yb_0.16_In_0.25_Zr_0.25_Cl_6_	Monoclinic C2/m	1.171 mS cm^−1^ @ RT	0.338 eV	[281]
High‐entropy halide electrolytes	Li_2.2_In_0.2_Sc_0.2_Zr_0.2_Hf_0.2_Ta_0.2_Cl_6_, HE‐5	Cubic close‐packed	4.69 mS cm^−1^ @30 °C	0.3 eV	[282]

**Figure 11:**
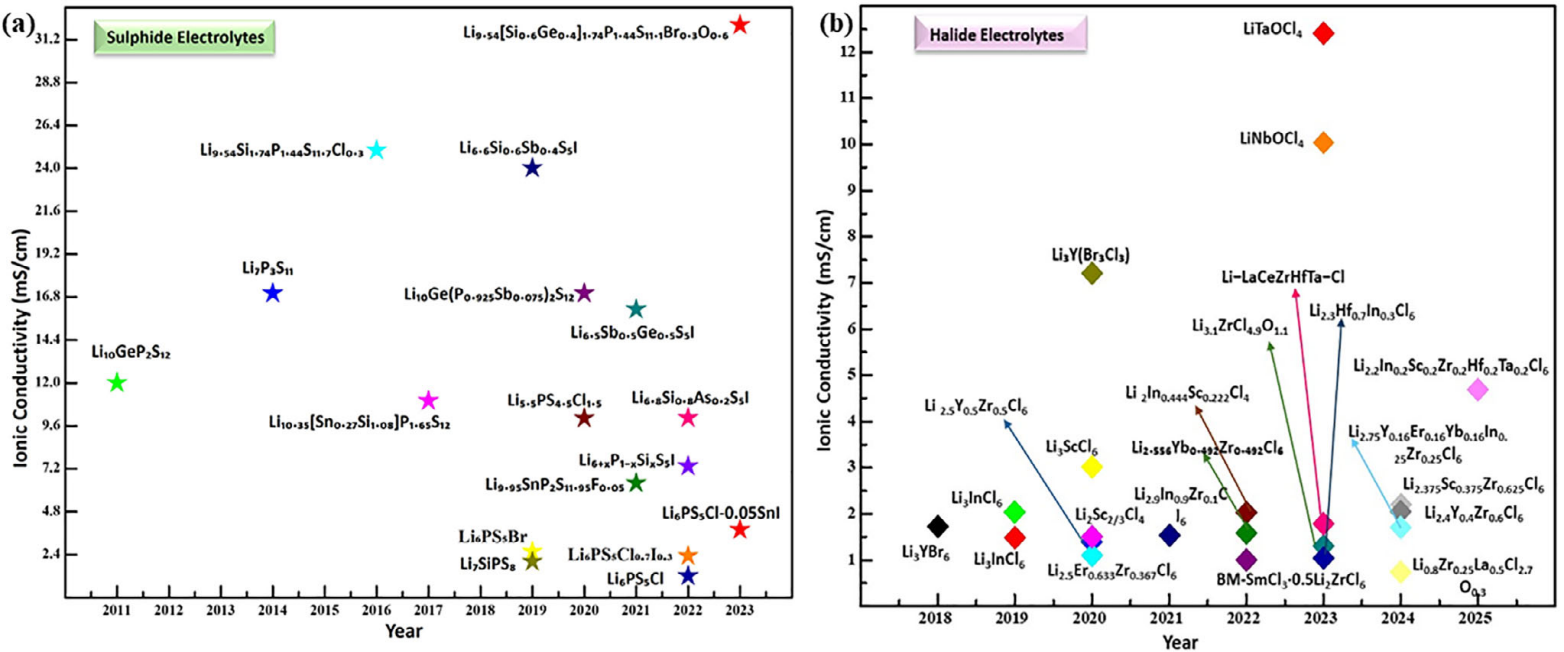
a) Progress in ionic conductivity of sulfide‐based solid electrolytes over time b) Advancements in halide‐based electrolytes: ionic conductivity trends from 2018.

Due to their highly polarizable S^2^^−^ anions,^[^ ^283^ ^]^ which form a soft lattice structure with lower Ea for Li migration, sulfide electrolytes have superior Li‐ion conductivity when compared to halide electrolytes, as shown in **Figure** **11**a. On the other hand, the halides (Cl^−^, Br^−^, I^−^, and F^−^) have higher migration barriers and stronger cationic bonds.^[^ ^223^ ^]^ In terms of structure, halides usually create dense, packed structures (hcp, ccp, monoclinic, or orthorhombic) that limit ion diffusion,^[^ ^284^ ^]^ whereas sulfides such as Li_7_P_3_S_11_, Li_10_GeP_2_S_12_, and argyrodite Li_6_PS_5_X feature open‐framework 3D conduction channels. Sulfides also accommodate cationic substitutions (Si, Sn, Ge, Sb, etc.), enhancing conductivity,^[^ ^285^ ^]^ whereas halides rely on Y^3^⁺, In^3^⁺, and Sc^3^⁺ substitutions, which can increase the Ea.^[^ ^286^ ^]^ The increased amount of Li in sulfides supports vacancy hopping and faster diffusion.^[^ ^287^ ^]^ In contrast, halides depend on cationic disorder or aliovalent doping, which is less efficient. Since 2018, developments in multi‐anion compositions (oxyhalides, high‐entropy halides) and aliovalent cation doping have greatly increased the conductivity of halide electrolytes, improving structural stability and Li‐ion transport.^[^ ^262^, ^282^ ^]^ Recent developments have reduced the gap with sulfide electrolytes by pushing ionic conductivities above 10 mS cm^−1^, especially in oxygen‐substituted halides (LiNbOCl_4_, LiTaOCl_4_).^[^ ^264^ ^]^ as shown in Figure 11b.

### 4.3 Synthesis and Electrochemical Stability

#### 4.3.1 Halide Electrolyte Synthesis via Solid‐State or Liquid‐Phase

**Figure 12:**
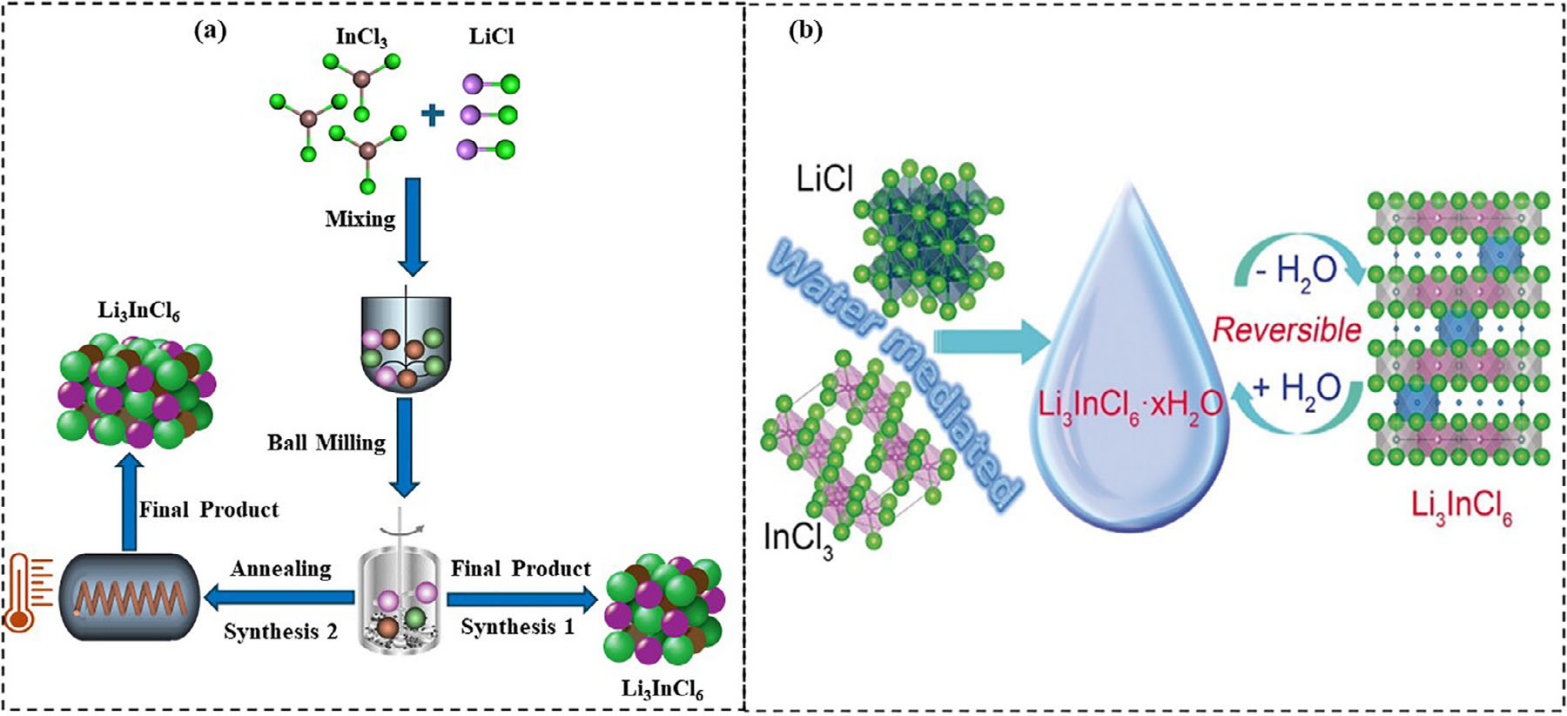
Synthesis routes a) solid‐state reactions b) Water‐mediated synthesis pathway for Li_3_InCl_6_ solid‐state electrolyte (SSE), highlighting the reversible transformation between the hydrated phase (Li_3_InCl_6_·x H_2_O) and the dehydrated Li_3_InCl_6_. Green represents Cl, purple represents In, and blue represents Li. Adapted with permission.^[^ ^268^ ^] Copyright 2019, Wiley‐VCH Verlag GmbH & Co. KGaA, Weinheim.^

Similar to other types of SEs, halide SEs can be produced through various synthesis routes. These include conventional solid‐state reactions such as ball milling and annealing, as well as combinations of these methods, as shown in **Figure** **12**a, or liquid‐phase synthesis techniques, as shown in Figure 12b.^[^ ^262^, ^288^ ^]^

As shown in **Table** **7**, solid‐state, mechanochemical, or solvent‐mediated (aqueous/ethanol) techniques are used to create halide electrolytes; ball milling introduces defects that improve ionic conductivity. Crystallinity is ensured by moderate post‐treatment (200‐550 °C), but over‐annealing may impair performance because of phase shifts or defect removal. Structural features like off‐stoichiometry, dopants (e.g., Zr, Fe, Yb), disorder, vacancies, and crystal orientation (e.g., (131) vs (001)) critically impact Li⁺ mobility. Leading materials have low electronic conductivity and >1 mS cm^−1^ conductivity at ambient temperature. The majority are stable up to 4.3–4.5 V vs Li⁺/Li; however, lifetime may require interface engineering. Ethanol and water‐based syntheses, unlike ampule‐based or high‐temperature methods, are scalable and cost‐effective. These featured excellent cycle stability with high voltage cathodes (e.g., NMC) retaining 90–95% of initial capacity post 200–1000 cycles, emphasizing the importance of appropriate synthesis, structure, and compatibility.

**Table 7:** Synthesis techniques and electrochemical performance of halide‐based solid electrolytes.

Halide electrolyte	Synthesis method	Temperature	Electrochemical properties	Voltage	Remark	Refs.
Li_3_MBr_6_ (M = Sm‐Lu, Y) monoclinic	Solid‐state reaction in quartz ampoule (LiBr + MBr_3_)	400 °C for 2 weeks (powder); melt at ≈600 °C for single crystals	High Li^+^ mobility; σ ≈ 0.01 S/cm at T >300 °C	–	This synthesis approach poses significant challenges for large‐scale industrial production	[212]
Li_2‐2x_Cd_1+x_Cl_4_ X = 0.05 Li_l.9_Cd_1.05_C1_4_ cubic	Solid‐state reaction: LiCl + CdCl_2_.	1 week just below melting point (≈480–500 °C)	Max σ ≈ 3.5 × 10^−1^ S/cm at 400 °C; Ea ≈ 54 kJ mol^−1^; multiple phase transitions observed	Decomposition potential ≈1.1 V	Spinel structure: Cd^2^⁺ substitution limited (x ≤ 0.05); highest conductivity at 400 °C; complex phase behavior	[289]
Li_3_YCl_6_ Trigonal	Mechanochemical	Ball‐milling + 5 min @ 550 °C (quenched)	Higher ionic conductivity, lower activation barrier	≈4 V vs Li⁺/Li	High Y‐disorder (43%), distorted Li substructure improves mobility	[290]
Li_3_YCl_6_ Trigonal	Ampule	1 week @ 550 °C (slow cooled)	Lower ionic conductivity, grain boundary effects	≈4 V vs Li⁺/Li	Low Y‐disorder (23%), less mobile Li, more grain boundary resistance	[290]
Li_3_ErI_6_	Ball milling + 5 min annealing at 550 °C in vacuum	−40–60 °C (impedance testing), synthesis at 550 °C	Ionic conductivity: 0.65 mS cm^−1^ (as‐milled), 0.39 mS cm^−1^ (annealed).	–	The ionic conductivity of Li_3_ErCl_6_ decreased following annealing	[227]
Li_2_ZrCl_6_	Mechanochemical milling of LiCl and ZrCl_4_	RT (as‐milled), annealed at 215 °C, 350 °C	Ionic conductivity: 0.81 mS cm^−1^ (as‐milled), 5.81 × 10^−6^ S cm^−1^ (annealed at 350 °C)	Electrochemical stability window: 1.75–4.25 V vs Li/Li⁺	The ionic conductivity of Li_2_ZrCl_6_ decreased following annealing	[291]
Li_2_Sc_2/3_Cl_4_	Solid‐state reaction	RT	Ionic conductivity: 1.5 mS cm^−1^; Ea: 0.34 eV; Stable cycling with high voltage cathodes	Up to 4.6 V vs Li/Li^+^	First spinel‐type superionic halide; Disordered structure with 3D Li^+^ diffusion pathways; High stability without cathode coating	[273]
Li_2.25_Zr_0.75_Fe_0.25_Cl_6_	Ball‐milling (4–16 h)	RT, up to 60 °C	Ionic conductivity: 0.74–0.80 mS cm^−1^ (milled), drops to mS cm^−1^ after annealing	3.0–4.3 V (stable), 3.0–4.5 V (degrades)	Short milling (4 h) gives decent conductivity, but 16 h milling improves capacity retention and rate performance. Annealing degrades performance due to phase transition.	[292]
Li_2.556_Yb_0.492_Zr_0.492_Cl_6_	High temperature melting and Zr doping	Annealing temperature	1.58 mS cm^−1^ ionic conductivity, stable up to 4.5 V, excellent moisture stability	Up to 4.5 V vs Li+/Li	High voltage stability from strong Zr‐Cl bonds and stable Yb‐based structure; excellent performance with NCM and LCO cathodes	[276]
Li_3_YBr_6_ (AN‐Li_3_YBr_6_)	Ball milling (550 rpm, 32 h) + Annealing	500 °C for 5 h	Ionic conductivity: 3.31 mS cm^−1^ at 30 °C	2.5–4.4 V vs Li⁺/Li	Improved crystallinity and Li⁺ mobility, enhanced by LiNbO_3_ coating	[293]
Li_3_InCl_6_	Water‐mediated synthesis + vacuum drying	Up to 200 °C (progressive heating)	Ionic conductivity (mS cm^−1^): HV: 2.70, LV: 0.96, Ar: 0.39, N_2_: 0.22	2.5–4.4 V vs Li⁺/Li	Oxy‐contaminants (In‐O species, crystal water) reduce conductivity; HV drying yields best performance; suitable for high‐capacity ASSLIBs.	[294]
Li_3_InCl_6_	Water‐mediated synthesis + vacuum dehydration	200 °C (vacuum, 4 h)	Ionic conductivity: 2.04 mScm^−1^ @ 25 °C	≈0–4.5 V (vs. Li/Li+)	High humidity stability with reversible hydration/dehydration. Compatible with NMC811. Scalable and low‐cost synthesis.	[268]
Li_2.61_Y_1.13_Cl_6_	Mechanochemical synthesis	RT / Annealed up to 400 °C	Ionic conductivity: 0.47 mS cm^−1^ @ 25 °C; Ea: ≈0.2 eV; Very low electronic conductivity	≈4.0 V vs Li/Li+	excellent cycling performance (90% retention after 1000 cycles); Li^+^ carriers thermally activated; Enhanced by defects and low migration barrier	[295]
Li_3_InCl_6_	Ethanol‐mediated dissolution and post‐treatment	200 °C for 3 h (post‐heating)	0.79 mS cm^−1^ @ 20 °C, Ea = 0.27 eV, good stability, recoverable after moisture exposure	Stable up to ≈4.25 V vs Li+/Li	Energy‐friendly synthesis; crystal orientation (131/001) affects conductivity; good compatibility with NMC811; 94.8% capacity retention after 200 cycles	[296]

#### 4.3.2 Halide Electrolyte Synthesis via DFT Approach

The development of a wide range of ab initio computational methods, especially the highly effective density functional theory (DFT) techniques,^[^ ^297^, ^298^ ^]^ facilitated the prediction of intrinsic properties of battery compounds, including their electrochemical stability, ionic conductivity, and interfacial compatibility. DFT calculations are essential in the prediction and optimization of solid electrolyte materials, generating atomic‐level descriptors that aid in extracting fundamental information on the mechanism of Li‐ion diffusion, Ea, and electrochemical stability windows.^[^ ^299^ ^]^ These techniques allow precise calculation of ionic conductivities (σ_i_), which is crucial for developing ASSB technologies when combined with ab initio molecular dynamics (AIMD) simulations.^[^ ^223^ ^]^ Li_2.5_Sc_0.5_Zr_0.5_Cl_6_ emerged as a top performer in the computational analysis, showing outstanding ionic conductivity (89 mS cm^−1^) and a low Ea of 0.16 eV, making it a strong contender for high‐efficiency ASSBs.^[^ ^300^ ^]^

**Figure 13:**
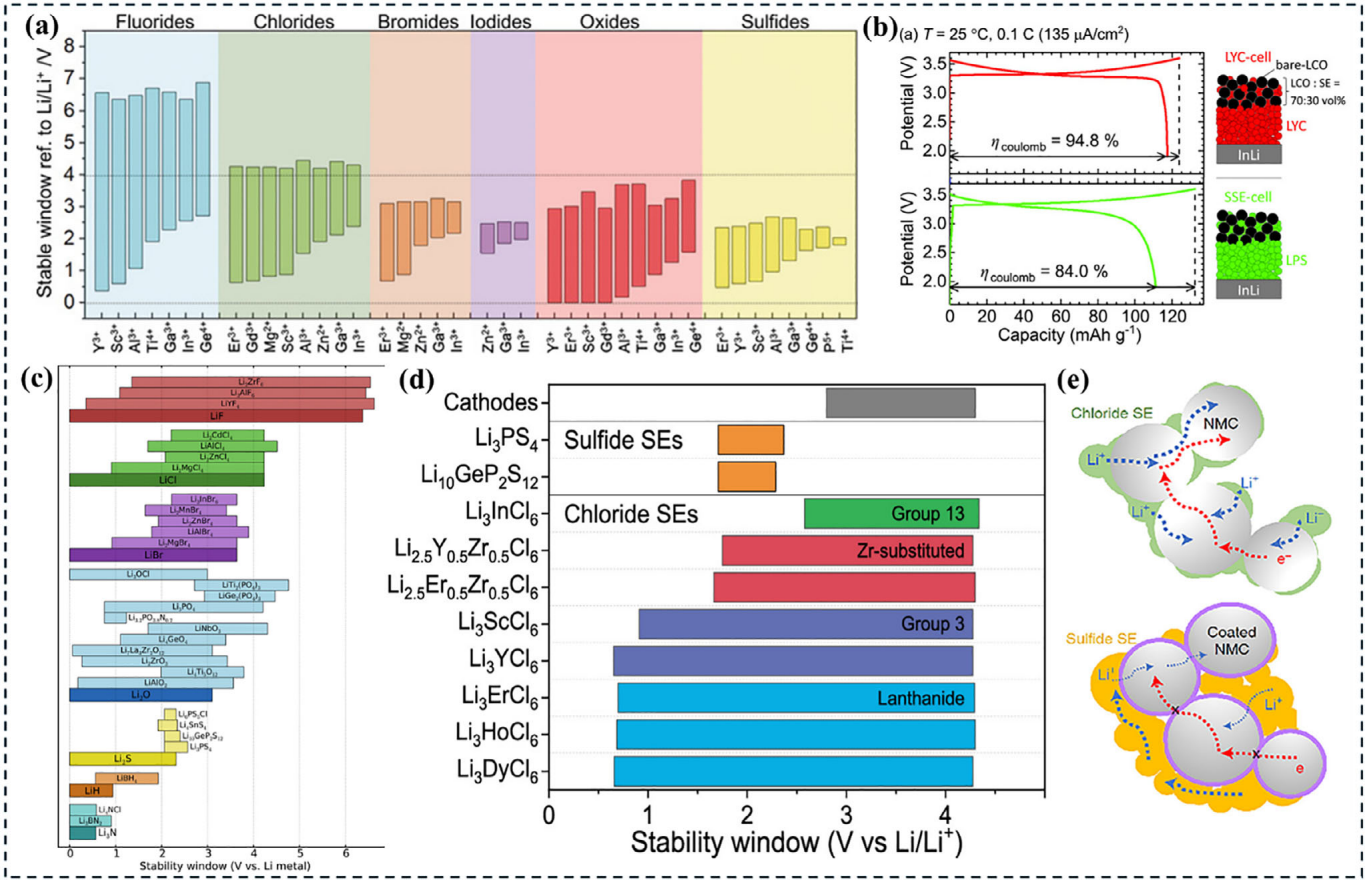
a) Thermodynamically calculated intrinsic electrochemical windows of Li‐M‐X ternary compounds, including fluorides, chlorides, bromides, iodides, oxides, and sulfides, where M represents a metal cation in its most common highest valence state. Adapted with permission.^[^ ^223^ ^]^ Copyright 2019, Wiley‐VCH Verlag GmbH & Co. KGaA, Weinheim; b) Initial charge–discharge profiles of bulk‐type ASSB cells using Li_3_YCl_6_ and Li_3_PS_4_ at 25 °C and 0.1 C. Adapted with permission.^[^ ^224^ ^]^ Copyrigt 2018, Wiley‐VCH Verlag GmbH & Co. KGaA, Weinheim; c) Electrochemical stability comparison of various electrolyte materials categorized by anion type. Adapted with permission.^[^ ^303^ ^]^ Copyright 2015, American Chemical Society; d) Electrochemical stability windows of Li chloride and sulfide solid electrolytes and the operating potentials of cathode materials. Adapted with permission.^[^ ^304^ ^]^ Copyright 2021, American Chemical Society; e) The diagram illustrating continuous Li‐ion transport pathways. Adapted with permission.^[^ ^145^ ^] Copyright 2023, Elsevier.^

In battery systems, the ability of a material, typically an electrolyte or electrode, to retain its chemical structure and functionality under the battery's operating voltage without breaking down or reacting is referred to as electrochemical stability ^[^ ^301^ ^]^. The wider the electrochemical stability window, the more suitable a material is as a solid electrolyte in batteries with high‐voltage cathodes or metal anodes.^[^ ^302^, ^303^ ^]^ As shown in **Figure** **13**a, halide‐based SEs demonstrate better chemical oxidation stability than sulfide SEs. Due to their strong electronegativity and oxidation resistance, fluorides have the widest electrochemical windows (up to ≈6–7 V), which makes them perfect for durable coatings or high‐voltage cathodes. Chlorides offer moderate ESWs (≈3.5–5 V), better than sulfides. At the same time, bromides and iodides show narrower ESWs, especially iodides (<3 V), due to lower oxidation potentials, making them easier to oxidize and hence less stable. With O^2^^−^ offering strong chemical stability, oxides generally provide stable voltage windows (≈3–5 V), making them widely used in SSEs like LLZO. Chlorides are more reactive than fluorides but less so than bromides or iodides. Sulfides, with ESWs around 2–3 V, rank among the most reactive, especially under high voltage. However, their superionic conductivity makes them valuable, despite lower stability. Chlorides and bromides, with intermediate behavior, are promising candidates for future halide‐based electrolytes. Bulk‐type ASSBs using Li_3_YCl_6_ SEs and uncoated LCO as the cathode active material demonstrated stable cycling performance and a high initial coulombic efficiency of 94.8%. Although Li_3_PS_4_ has excellent bulk ionic conductivity, its interfacial instability with oxide cathodes like LCO severely limits its performance in full cells, making chloride‐based SEs (like Li_3_YCl_6_) far more suitable for practical ASSB applications (Figure 13b). Primarily, electrolyte stability is governed by the anion chemistry. While halides (F^−^, Cl^−^) offer high oxidation stability and wider windows, sulfides and phosphides have low anodic limits, making them more reactive with high‐voltage cathodes (Figure 13c). All of the chloride SEs have high oxidation potentials of ≈4.3 V vs Li/Li⁺, as seen in Figure 13d, which suggests robust oxidative stability. On the other hand, sulfide SEs like Li_3_PS_4_ and Li_10_GeP_2_S_12_ break down at significantly lower oxidation potential (≈2.4 V). Despite having a high ionic conductivity, they are susceptible if appropriate mitigating measures are not taken because of their weak oxidative stability. Direct contact with NMC cathodes is made possible by chloride SE's stable Li ion transport and great oxidative stability, which eliminates the need for protective coatings and major degradation. In contrast, sulfide SEs require cathode coatings to prevent interfacial degradation due to their lower oxidative stability. Without coatings, direct contact with NMC would oxidize the sulfide SE, forming a resistive interphase. While Li ions can still migrate across the interface, this setup introduces additional interfacial resistance and processing complexity to maintain stability (Figure 13e).

According to **Table** **8**, halide SEs, like Li_2_In_1/3_Sc_1/3_Cl_4_ and Li_2.4_Y_0.4_Zr_0.6_Cl_5.85_F_0.15_, are perfect for high‐voltage cathodes because of their exceptional cycle life, rate performance, and environmental stability. However, to prevent degradation at the Li or In anode interface, interlayers such as LGPS are necessary.^[^ ^268^ ^]^ Sulfide SSEs provide high ionic conductivity and strong compatibility with Li metal despite their lower oxidative stability. Cathode‐side stability requires coatings or dopants.^[^ ^313^ ^]^ Sulfides are preferred for anode‐side performance and quick Li ion transfer,^[^ ^314^ ^]^ whereas halides are preferred for cathode‐side durability.^[^ ^303^ ^]^ Halide SEs, particularly those based on chloride, such as Li_3_InCl_6_ and Li_2_In_0.444_Sc_0.222_Cl_4_, provide a potent combination of high ionic conductivity (≈2 × 10^−3^ Scm^−1^), high voltage stability (>4 V vs Li/Li⁺), and outstanding compatibility with high‐voltage cathodes without the need for coatings. These SEs make low interfacial resistance, reliable long‐term cycling, and effective 3D Li‐ion routes possible. Performance is further improved by employing aliovalent doping and optimizing the central metal cations (such as In^3^⁺ and Sc^3^⁺).^[^ ^145^ ^]^ Dual‐halide systems with fluorine are perfect for high‐energy, room‐temperature SSBs because they increase the electrochemical window up to 7.0 V.^[^ ^305^ ^]^

**Table 8:** Comparison of halide and sulfide SEs: Ionic Conductivity, Stability, and Cycling Performance in All‐Solid‐State Li Cells.

Cell configuration	Electrolyte information	Active material loading/wt%	First discharge capacity/ coulombic efficiency	Cyclability and rate capability (retention)	Refs.
Li‐In / Li_3_YCl_6_ / LiCoO_2_ (LYC‐cell)	Li_3_YCl_6_ (LYC): Halide SSE; σ_i_ = 0.51 mS cm^−1^, σ_e_ = 2.8 × 10^−9^ S cm^−1^; 4V cathode‐stable; deformable.	9.9 mg cm^−2^ (LCO)	Coulombic efficiency: 94.8%	98% capacity retention after 100 cycles @ 0.1 C 40% capacity retention @ 5 C 99.9% coulombic efficiency after 10 cycles	[224]
LiIn / Li_3_InCl_6_/ LiCoO	Li_3_InCl_6_: Halide SSE; σ_i_ = 1.49 mS cm^−1^, σ_e_ = 5.4 × 10^−9^ S cm^−1^; air‐stable; compatible with oxide cathodes up to 4.2 V	100 µm cathode, 320 µm SSE layer	127 mAh g^−1^, (92%)	95 mAh g^−1^ after 100 cycles @ 0.1 C; rate capability: 127, 125, 120, 111, and 97 mAh g^−1^ at 0.1, 0.2, 0.5, 0.8, 1 C, respectively	[267]
Li‐In / LPSCl‐Li_2_ZrCl_6_ / NMC811	Li_2_ZrCl_6_: Halide SSE; σ_i_ = 0.81 mS cm^−1^, σ_e_ = 9.22 × 10^−^⁸ S cm^−1^; moisture‐resistant; cost‐effective ($1.38 m^−2^)	–	181 mAh g^−1^, (90.3%)	149 mAh g^−1^ after 200 cycles @1 C; 176, 96 mAh g^−1^ @0.2, 2 C	[291]
Li‐In / Li_2.25_Zr_0.75_Fe_0.25_Cl_6_ / LiCoO_2_	Li_2.25_Zr_0.75_Fe_0.25_Cl_6_; σ_i_ = 0.98 mS cm^−1^ @30 °C; good air stability; high interfacial stability; mechano‐chemically prepared	LiCoO_2_:SE:C = 70:30:3 wt%	162 mAh g^−1^ (90.5%)	Slightly degraded cycling compared to Li_2_ZrCl_6_(91.4%); better rate capability due to higher Li⁺ conductivity	[225]
Li / Li_2_In_1/3_Sc_1/3_Cl_4_ / NCM85	Li_2_In_1/3_Sc_1/3_Cl_4_: Halide SSE; σ_i_ = 2.0 mS cm^−1^, σ_e_= 4.7 × 10^−10^ S cm^−1^; stable up to 4.8 V vs Li⁺/L	21.59 mg cm^−2^	192 mAh g^−1^	>3000 cycles, 80% retention @ 3 C; 95% retention @ 110 cycles (4.8 V); >4 mAh cm^−2^ capacity with high loading	[275]
Li / Li_5.3_PS_4.3_ClBr_0.7_ / Li_2.4_Y_0.4_Zr_0.6_Cl_5.85_F_0.15_ / NCM811	Li_2.4_Y_0.4_Zr_0.6_Cl_5.85_F_0.15_: Halide SSE; σ_i_ = 1.45 mS cm^−1^, σ_e_ = 2.33 × 10^−9^ S cm^−1^; stability window: 1.29–3.9 V (vs Li⁺/Li); moisture‐tolerant	5 mg cm^−2^	190 mAh g^−1^ (87%)	182 mAh g^−1^ after 100 cycles @ 0.1 C (95.1%); 128 mAh g^−1^ after 250 cycles @ 0.5 C (95.5%); 140 mAh g^−1^ @ 1 C; 110 mAh g^−1^ @ 2 C; 78 mAh g^−1^ at 5 C	[279]
In / Li_6_PS_5_Cl / Li_3_InCl_6_ / Li_3_InCl_4.8_F_1.2_ / LCO	Li_3_InCl_4.8_F_1.2_: Halide SSE; σ_i_ = 0.51 mS cm^−1^, σ_e_ = 1.02 × 10^−9^ S cm^−1^; anodic stability >6 V; dense morphology	‐	160.6 mAh g^−1^ (92%)	102 mAh g^−1^ after 70 cycles @ 4.47 V; 203.7 mAh g^−1^ @4.8 V	[305]
Li−In/Li_6.7_Si_0.7_Sb_0.3_S_5_I/ Li_2.7_Yb_0.7_Zr_0.3_Cl_6_/NCM622 or LCO	Li_2.7_Yb_0.7_Zr_0.3_Cl_6_: halide SSE; σ_i_ = 1.1 mS cm^−1^, σ_e_ ≈ 1 × 10^−10^ S cm^−1^; E_a_ = 0.30 eV; stable 2.8–4.3 V (vs Li⁺/Li)	cathode: SE = 8:2 weight ratio	LCO: ≈125 mAh g^−1^ @ 0.1 C NMC622: ≈170 mAh g^−1^ @ 0.2 C	LCO: ≈90% retention after 200 cycles @ 0.5 C NMC622: ≈80% retention after 150 cycles @ 0.2 C	[306]
Li / Li_3_YBr_5.7_F_0.3_/ LCO@LIC	Li_3_YBr_5.7_F_0.3_: Halide SSE; σ_i_ = 1.8 mS cm^−1^, σ_e_ = 4.77 × 10^−9^ S cm^−1^; E_a_ = 0.39 eV; Li‐stable via in situ LiF/YF_x_ interfacial layer	10 mg cm^−2^	121.6 mAh g^−1^ (90%)	≈60% retention over 70 cycles at ≈0.14 mA cm^−2^; stable plating/stripping for 1000 h at 0.75 mA cm^−2^	[307]
Li‐In / LGPS / HLPO@NMC811	Li_10_GeP_2_S_12_: Sulfide SSE; σ_i_ = 10^−2^–10^−4^ S cm^−1^; dual‐layer ALD Li_3_PO_4_ coated cathode; mitigates side reactions and cracking	NMC811 and LGPS in a 70:30 weight ratio	170.6 mAh g^−1^ (75.1%)	96.1 mAh g^−1^ after 300 cycles at 0.2 C (77.9% retention); 115 mAh g^−1^ at 1 C	[308]
Li / Li_6_PS_5_Cl / LNO‐coated NCA	Li_6_PS_5_Cl; sulfide‐based electrolyte; used with 5 MPa stack pressure	3.55 mg cm^−2^	150 mAh g^−1^ (69%)	80.9% retention after 100 cycles at C/10; 229 cycles total	[309]
Li−In/Li_6_PS_5‐x_O_x_Br/NCM811	Li_6_PS_4.7_O_0.3_Br: O^−^doped argyrodite; σ_i_ ≈ 1.4 mS cm^−1^; enhanced dendrite suppression and interfacial stability	15 mg (composite electrode)	106 mAh g^−1^ (47%)	108.7 to 47.4 mAh g^−1^ from 0.1 C to 0.8 C; 92 stable cycles with Li metal, without short circuiting	[310]
Li / LiI–Li_3_PS_4_ / NCM622	LiI‐doped Li_3_PS_4_: Sulfide SSE; σ_i_ >1 mS cm^−1^; two‐step ball milled and annealed at 160 °C	≈7 mg cm^−2^	170.1 mAh g^−1^ (87.1%)	74.3% retention after 40 cycles at 0.1 C; stable at 0.5 C	[104]
Li_4_Ti_5_O_12_/Li_10_SnP_2_S_12_/NCM811	Li_10_SnP_2_S_12_ (LSPS): Sulfide SSE; σ_i_ = 2.7 mS cm^−1^	10.2 mg cm^−2^	187 mAh g^−1^ (74%)	64.5% after 100 cycles at 0.1 C. Exceptional rate capability (102 mAh g^−1^ at 180 mA g^−1^)	[311]
Li–In / HRLA (Li_5.5_PS_4.5_Cl_1.5_) / Nb‐NCM	Li_5.5_PS_4.5_Cl_1.5_ (HRLA): Sulfide SSE; σ_i_ = 10.2 mS cm^−1^, σ_e_ ≈1 × 10^−^⁵ mS cm^−1^; ESW = 1.8–2.5 V (vs Li⁺/Li)	‐	149.7 mAh g^−1^ (first cycle)	72.1% capacity retention after 60 cycles; 93.1% retention on returning to 0.1 C	[312]

### 4.4 Mechanical Properties

Mechanical stability is the capacity to maintain one's integrity under changes in internal or external stress. The stress could be caused by the electrode/electrolyte interface or the unavoidable volume change in the composite electrode.^[^ ^6^ ^]^ During cycling, SSBs have been shown to exhibit dendrite development, cracking, and pulverization, raising serious concerns about mechanical instability in ASSBs.^[^ ^315^ ^]^ Sulfide electrolytes offer a balanced advantage in mechanical properties; they are more flexible and processable than oxides and stiffer than polymers. They are not the strongest, but they are likely the most practical for battery integration due to their cold‐pressing ability and decent stiffness.^[^ ^27^ ^]^ Li_3_YCl_6_ and Li_6_PS_5_Cl_0.5_Br_0.5_ differ significantly as SEs for ASSBs. Li_3_YCl_6_, which is a halide‐based SE, is more stable and can handle higher voltages without breaking down. On the other hand, Li_6_PS_5_Cl_0.5_Br_0.5_, which is a sulfide‐based electrolyte, fails at high voltages and produces unwanted substances like P_2_S_5_, PO_4_^3−^, and SO_4_^2−^. Mechanically, Li_3_YCl_6_ is rigid and dense with a higher specific density of 2.43 gcm^−3^, needing more material, no less than 40% by weight, to cover the cathode well and avoid cracking. In contrast, Li_6_PS_5_Cl_0.5_Br_0.5_ is softer and less dense (1.96 gcm^−3^), allowing improved mechanical compliance and particle contact at lower loadings. However, it suffers from volumetric shrinkage and structural degradation due to side reactions. Both can prevent cracking in a single NCA when appropriately used, but Li_3_YCl_6_ offers longer‐term stability with an optimized design.^[^ ^316^ ^]^ Tao et al. created a new type of viscoelastic inorganic glass SEs (VIGLAS) by partially replacing the chlorine with oxygen in melted salts like LiAlCl_4_ and NaAlCl_4_. The resulting materials, LiAlCl_2.5_O_0.75_ and NaAlCl_2.5_O_0.75_, exhibit high ionic conductivity and polymer‐like viscoelasticity. They also exhibit superior chemo‐mechanical compatibility with 4.3 V cathodes, enabling pressure‐less operation in SSBs.^[^ ^317^ ^]^ To accept stress during battery cycling, the majority of halide SEs use close‐packed anion frameworks, either hcp or ccp, which provide moderate mechanical deformability and low energy barriers for Li ion diffusion. Among these, ccp structures like Li_3_InCl_6_ typically show better mechanical integrity than hcp structures like Li_3_YCl_6_, particularly in hot or humid environments. Strategies for doping and defect engineering are used to improve the mechanical robustness further. Anion doping (e.g., with F or O) and cation doping (e.g., with In, Zr, or Hf) aid in the creation of partially amorphous or defect‐rich structures that are better able to absorb strain. While modifications to composition alter unit cell dimensions and bonding interactions, purposefully introducing oxygen brings about a form of controlled disorder highly useful for flexibility and crack inhibition over time.^[^ ^318^ ^]^

### 4.5 Scaleup Technology and Safety

Halide SEs are emerging as a class of scale‐up‐friendly materials. Their promising balance of performance, processability, and cost‐effectiveness is paving the way for the mass commercialization of ASSBs.^[^ ^319^ ^]^ Halide‐based SSEs are growing to become promising candidates for scalable ASSB technologies due to their high ionic conductivity (up to 7 mS cm^−1^), such as Li_3_Y(Br_3_Cl_3_) with 7.2 mS cm^−1,[^ ^277^ ^]^ LiNbOCl_4_ with 10.04 mS cm^−1^, and LiTaOCl_4_ with 12.4 mS cm^−1,[^ ^264^ ^]^ as well as their voltage stability, mechanical deformability, and compatibility with high‐voltage cathodes like single‐crystalline NMC811.^[^ ^311^ ^]^ Commercial‐scale deployment is possible with leading materials like Li_3_InCl_6,_^[^ ^267^, ^268^ ^]^ Li_3_YCl_6_^[^ ^224^, ^320^, ^321^ ^]^ and Li_3_ScCl_6,_^[^ ^269^ ^]^ as well as less expensive substitutes like Li_2_ZrCl_6,_^[^ ^225^ ^]^ particularly when aliovalent substitution is employed to improve performance and decrease expenses.^[^ ^291^ ^]^ An ASSB using an NCM811 cathode, LiIn anode, and a very thin 25 µm Li_3_InCl_6_/ZrO_2_ halide inorganic solid electrolyte (LZ‐ISE) membrane made through an easy solution infusion method shows a specific energy of ≈350 Wh kg^−1^, a reversible capacity of 131.2 mAh g^−1^, and keeps over 80% of its capacity after 200 cycles. The process works at low temperatures (80–200 °C) without needing sintering or ball milling the materials, producing thin, flexible, heat‐resistant (up to 400 °C), pressure‐resistant, non‐flammable, and electrochemically durable membranes.^[^ ^322^ ^]^ Dry coating technologies could also be a viable option for SEs.^[^ ^323^ ^]^ Dry processing makes ASSBs safer by removing flammable solvents, improving the flow of ions to reduce internal resistance and minimize localized heating, and strengthening them to lower the chances of short circuits if they get physically damaged.^[^ ^324^ ^]^ Dry‐processed Li_3_InCl_6_‐PTFE films (especially Li_3_InCl_6_‐0.5PTFE) demonstrate a scalable, moisture‐stable, and mechanically robust alternative for halide SEs in ASSBs. The method offers a viable transition from lab to production, though interfacial compatibility (e.g., with Li_6_PS_5_Cl) remains an area for further study.^[^ ^325^ ^]^ The HELENA partnership is advancing Li‐metal halide‐based SSB with a focus on material innovation, sustainability, and scalable manufacturing. Important progress includes using new halide SEs like Li_3_YBr_6_, Li_3_YBr_2.1_Cl_3.9_, and Li_3_Y_1−x_M_x_Br_2_Cl_4_, which can conduct ions with high efficiency at rates up to 5.1 mS cm^−1^ and remain stable in normal conditions. The cathode technology is moving from NMC622 to NMC811 to increase energy density (up to 210 mAh g^−1^), and this is improved by adding surface coatings for better stability at the interface. On the anode side, a thin layer of Li metal (less than 40 µm) is used along with specially designed SEI layers to prevent dendrites and lower resistance at the interface. These innovations target EV applications requiring ≥450 Wh kg^−1^ energy density, 750 full cycles at 70% SOH, and fast charging from 10% to 80% in under 20 min.^[^ ^326^ ^]^ To further expand the use of halide SEs into Li‐S,^[^ ^327^ ^]^ Li‐Se,^[^ ^328^ ^]^ Na‐ion,^[^ ^329^ ^]^ and organic battery systems for next‐generation energy storage,^[^ ^330^ ^]^ future scale‐up efforts must prioritize low‐cost, earth‐abundant elements (such as Y, Zr, La, and Ce), the development of moisture‐tolerant electrolytes, dry‐processable films, and composite/hybrid SSEs.^[^ ^331^ ^]^

**Figure 14:**
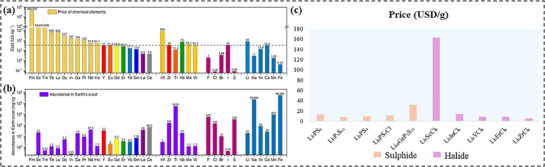
a) Price of Chemical Elements Used in Solid‐State Electrolytes (USD per kg); b) Abundance of Chemical Elements Used in Solid‐State Electrolytes in Earth's Crust (mg kg^−1^).^[^ ^331^ ^]^ c) Comparison of Halide and Sulfide Solid Electrolytes: Composition and Cost per Gram, data was drawn from.^[^ ^288^ ^] Copyright 2022, Wiley‐VCH GmbH.^

As shown in **Figure** **14**a,b, the high average cost of halide SE is largely driven by the use of Sc and In, both of which are costly and extremely scarce. Elements like Zr, Y, and Er offer a more balanced cost‐abundance profile, contributing to a relatively lower cost in some halide compositions. Cl is both cheap and abundant, making it a non‐issue cost‐wise in halide SEs.^[^ ^331^ ^]^ Halide‐based SEs are significantly pricier on average ≈2.8 times higher in price than sulfide‐based ones,^[^ ^288^ ^]^ as shown in Figure 14c.

### 4.6 Advantages and Challenges

**Figure 15:**
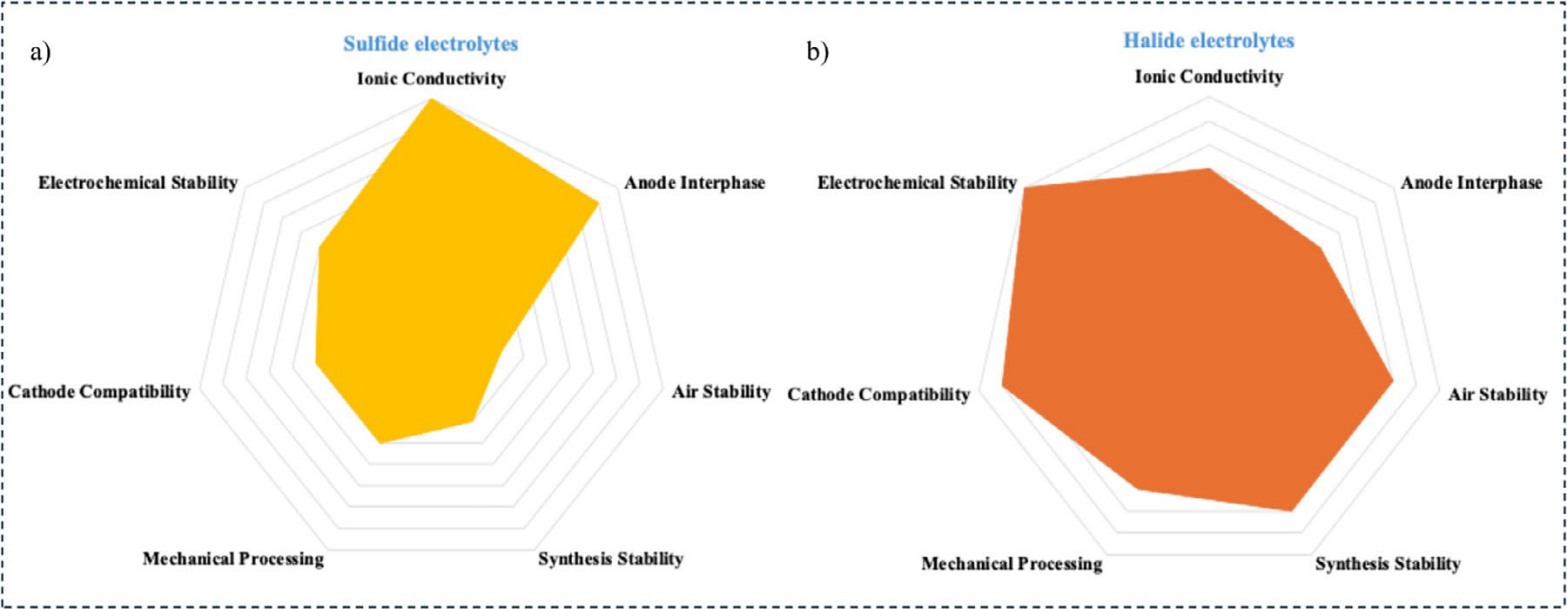
a) Radar Chart of Sulfide Electrolytes Performance in Lithium Solid‐State Batteries.b) Radar Chart of Halide Electrolytes Performance in Lithium Solid‐State Batteries.

Halide electrolytes have a number of significant materials, interface, and engineering‐related issues. Halide SEs are also usually hygroscopic. Many halide SEs, particularly chlorides and bromides, are hygroscopic due to their ionic bonds and the polarizability of halide anions. This tendency can cause hydration, structural damage, and a drop in ionic conductivity. In terms of materials, they suffer from poor environmental stability, including air sensitivity, relatively low ionic conductivity, and high costs. Interface issues can impact performance and safety and include resistance to charge transfer, poor solid‐material contact, mechanical stress, and Li dendritic development. From an engineering perspective, problems such as the requirement of high pressure to assemble parts, complicated integration of SSEs, matching chemicals, and the inefficiency of large‐scale roll‐to‐roll manufacturing make it harder to use these technologies in practical applications. Addressing these challenges is crucial for advancing halide‐based SSEs. Halide electrolytes offer several compelling advantages for ASSBs. First, they have high ionic conductivity, with materials like Li_3_YCl_6_ and Li_3_InCl_6_ showing conductivities at RT of ≈10^−3^ S cm^−1^, which is like or better than sulfide‐based electrolytes.^[^ ^224^ ^]^ Advanced amorphous systems such as xLi_2_O‐TaCl_5_ have reached ≈6.6 × 10^−3^ S cm^−1^, rivalling crystalline conductors.^[^ ^332^ ^]^ This performance improves with the addition of different types of ions (like In, Sc, Zr for cations and O^2‐^, F^−^ for anions), which create disorder in the structure, making it easier for Li ions to be transported around.^[^ ^333^ ^]^ Amorphization further eliminates grain boundary resistance, ensuring uniform Li ion transport with low Ea.^[^ ^318^ ^]^ Second, halide electrolytes can handle a wide range of voltages, usually over 4 V for chlorides^[^ ^223^ ^]^ and over 6 V for fluorides,^[^ ^334^ ^]^ making them suitable to use directly with high‐voltage cathodes (like LCO and NCM) without needing extra protective layers. F^−^doping further extends the oxidative stability by forming stable interphases like LiF.^[^ ^305^ ^]^ Third, they show better compatibility with regular and high‐capacity cathodes because they have low reaction energies (<100 meV atom^−1^), lower than those of sulfide electrolytes, allowing them to connect directly and form composite cathodes without needing coatings, which makes manufacturing easier.^[^ ^318^ ^]^ Fourth, halides offer excellent mechanical properties; their inherent softness and deformability improve solid‐solid contact and mechanical integrity under cycling.^[^ ^335^, ^336^ ^]^ Scalable ways to prepare them, like liquid‐phase processing and in situ growth (for example, Li_3_InCl_6_, LCO), help create large, efficient designs that have low resistance and high energy density.^[^ ^337^ ^]^ Fifth, halides show better air stability than sulfides. Materials like Li_2_ZrCl_6_^[^ ^291^ ^]^ and Li_3_InCl_6_ display reversible hydration behavior,^[^ ^267^ ^]^ while In‐doped Li_3_YCl_6_ resists moisture uptake by forming stable structures.^[^ ^270^, ^275^ ^]^ Surface coatings (e.g., Al_2_O_3_)^[^ ^338^ ^]^ or F^−^doping enhance humidity resistance further. Finally, halide electrolytes form stable interfaces with Li‐In and other alloy anodes. Unlike sulfides, they enable slow interfacial reactions and develop passivating layers like LiCl, mitigating degradation.^[^ ^339^ ^]^ For instance, Li_0.388_La_0.238_Ta_0.475_Cl_3_ demonstrates stable cycling and gradient interfacial behavior, confirming its potential for long‐term, high‐performance battery systems.^[^ ^263^ ^]^ **Figure** **15**a shows that sulfide electrolytes have ionic conductivity and lithium‐metal anode compatibility but suffer from cathode and air stability. In contrast, Figure 15b highlights that halide electrolytes offer greater electrochemical stability along with superior cathode and air compatibility. Overall, sulfides are more suitable for lithium metal anodes, while halides are better suited for high‐voltage cathodes.

## 5 Conclusion

To make SE‐based ASSB manufacturing work, we need to study the materials, electrodes, and cell designs together in science and engineering. To unlock the commercial promise of ASSBs, future research must move beyond material silos. Sulfides and halides are not rivals but partners; together, they form the foundation for multifunctional, high‐performance SSBs. The integration of smart materials, interface science, green chemistry, and industrial process design will be the cornerstone of the next leap in battery innovation. Future research strategy for ASSBs will include a combination of sulfides and halides.

1. Hybrid electrolyte design: Combining sulfides, which are effective with Li‐metal interfaces, and halides, which are stable in high‐voltage cathodes, creates multi‐layer or composite SEs that take advantage of both the materials.
2. Interface engineering improves battery stability by adding protective layers (like LiF and LiCl) to minimize degradation, while tiny structures and buffer layers decrease resistance and stop dendrite growth.
3. Conductivity enhancement focuses on improving the ion transport through doping, defect engineering, and mixed‐anion frameworks, while also exploring oxyhalides and amorphous structures to achieve high conductivity and stability.
4. Scalable manufacturing means creating ways to make halides at normal conditions and sulfides at low temperatures, focusing on dry processing, tape casting, and using solvents and water to allow for large‐scale production.
5. Sustainability efforts focus on replacing rare metals like In and Ge with more abundant elements such as Zr and Fe while also designing eco‐friendly, recyclable materials and manufacturing processes.
6. Digital tools and AI are leveraged to accelerate material discovery through simulations. At the same time, in situ diagnostics are applied to monitor interfaces and degradation processes in real time, enabling a deeper understanding and optimization of ASSBs.

Combining sulfides and halides to engineer smart interfaces, scale green production, and drive discovery with AI will provide a pathway to make safe, high‐performance, and cost‐effective ASSBs a commercial reality.

## Conflict of Interest

The authors declare no conflict of interest.
